# Cyclin E1 and RTK/RAS signaling drive CDK inhibitor resistance via activation of E2F and ETS

**DOI:** 10.18632/oncotarget.2673

**Published:** 2014-12-22

**Authors:** Barbie Taylor-Harding, Paul-Joseph Aspuria, Hasmik Agadjanian, Dong-Joo Cheon, Takako Mizuno, Danielle Greenberg, Jenieke R. Allen, Lindsay Spurka, Vincent Funari, Elizabeth Spiteri, Qiang Wang, Sandra Orsulic, Christine Walsh, Beth Y. Karlan, W. Ruprecht Wiedemeyer

**Affiliations:** ^1^ Women's Cancer Program at the Samuel Oschin Comprehensive Cancer Institute, Cedars-Sinai Medical Center, Los Angeles, CA 90048, USA; ^2^ Graduate Program in Biomedical Sciences and Translational Medicine, Cedars-Sinai Medical Center, Los Angeles, CA 90048, USA; ^3^ Genomics Core, Cedars-Sinai Medical Center, Los Angeles, CA 90048, USA; ^4^ Department of Pathology, Cedars-Sinai Medical Center, Los Angeles, CA 90048, USA; ^5^ Department of Medicine, Cedars-Sinai Medical Center, Los Angeles, CA 90048, USA; ^6^ Department of Obstetrics and Gynecology, David Geffen School of Medicine, University of California, Los Angeles, CA 90048, USA

**Keywords:** Cyclin-dependent kinase inhibitors, palbociclib, dinaciclib, cyclin E1, ovarian cancer

## Abstract

High-grade serous ovarian cancers (HGSOC) are genomically complex, heterogeneous cancers with a high mortality rate, due to acquired chemoresistance and lack of targeted therapy options. Cyclin-dependent kinase inhibitors (CDKi) target the retinoblastoma (RB) signaling network, and have been successfully incorporated into treatment regimens for breast and other cancers. Here, we have compared mechanisms of response and resistance to three CDKi that target either CDK4/6 or CDK2 and abrogate E2F target gene expression. We identify *CCNE1* gain and *RB1* loss as mechanisms of resistance to CDK4/6 inhibition, whereas receptor tyrosine kinase (RTK) and RAS signaling is associated with CDK2 inhibitor resistance. Mechanistically, we show that ETS factors are mediators of RTK/RAS signaling that cooperate with E2F in cell cycle progression. Consequently, CDK2 inhibition sensitizes cyclin E1-driven but not RAS-driven ovarian cancer cells to platinum-based chemotherapy. In summary, this study outlines a rational approach for incorporating CDKi into treatment regimens for HGSOC.

## INTRODUCTION

The term ovarian cancer describes a set of diseases with vastly differing tumor biology, signature genetic aberrations, and associated outcomes [1]. Within this complex, high-grade papillary serous ovarian cancer (HGSOC) is the most common and aggressive form [2], accounting for the majority of ovarian cancer-related deaths. HGSOC is characterized by *TP53* mutation and a high number of DNA copy number aberrations targeting known oncogenes (e.g. *MYC* 30%, *CCNE1* 20%, *MECOM* 25%, *KRAS* 11%) and tumor suppressor genes (e.g. *PTEN* 7%, *RB1* 8%) [2]. Since the introduction of platinum and taxane combination therapy, the five-year survival rate for ovarian cancer has been largely stagnant over several decades and remains only around 40% [3], rendering ovarian cancer the leading cause of death among gynecologic malignancies. Thus, there is a dire need for novel therapeutic strategies to improve HGSOC outcome.

Here, we have taken a systematic approach to assess cyclin-dependent kinase inhibitors (CDKi) for their potential in HGSOC treatment. CDKi target the retinoblastoma signaling pathway [4, 5], one of the most frequently altered signaling networks in HGSOC [2] and other cancers [6]. Therefore, CDKi could potentially benefit a large number of patients. However, early generation CDKi, such as Flavopiridol, failed in the clinic. Recently, two CDKi with different target spectra have entered phase 3 clinical trials in human cancer. PD0332991 (palbociclib), a specific inhibitor of CDK4 and CDK6 (CDK4/6) [7], shown to induce proliferation arrest and senescence in several different cancer types [8–11], was labeled a break through drug by the FDA in 2013 for its promising activity in estrogen receptor-positive breast cancer when combined with the aromatase inhibitor, letrozole. Similarly, the CDK1 and CDK2 (CDK1/2) inhibitor dinaciclib [12] entered a phase 3 trial in chronic lymphocytic leukemia.

Interphase CDK phosphorylate and inactivate the RB tumor suppressor protein and related pocket proteins, p107 (*RBL1*) and p130 (*RBL2*) [13]. This allows activator E2F transcription factors to transcribe genes required for G_1_-S transition, as well as DNA repair genes, such as *BRCA1* [14]. CDK require specific cyclin binding partners for their activity: E-type cyclins (cyclin E1, *CCNE1*; cyclin E2, C*CNE2*) bind to and activate CDK2, whereas the D-type cyclins (cyclin D1, *CCND1*; cyclin D2, *CCND2*; cyclin D3, *CCND3*) specifically activate CDK4/6. Accumulating evidence suggests that cyclin E-CDK2 signaling is an important driver of HGSOC proliferation. First, *CCNE1* (20%), *CCNE2* (3%) and *CDK2* (3%) are frequently amplified in HGSOC [2]. Second, both cyclin E1 and CDK2 were identified in a genome-wide shRNA screen as potential lineage-specific requirement genes [15]. Third, deregulated cyclin E1 can transform *Trp53*-mutant fallopian tube epithelial cells [16], which can give rise to HGSOC [17]. Though cyclin D genes are less frequently amplified in HGSOC (*CCND1*: 4%, *CCND2* 6%, *CCND3* 3%), cyclin D is downstream of and required for the oncogenic activity of RAS, MYC and ERBB2 [18–20]. Therefore, cyclin D and cyclin E may be differentially required in different subsets of HGSOC, indicating that CDK4/6 inhibitors and CDK1/2 inhibitors may be most effective in distinct responder populations.

We have directly compared the response and resistance mechanisms for CDK4/6 inhibition (PD0332991) and CDK2 inhibition (SNS032 [21]; dinaciclib) in a panel of ovarian cancer cell lines. Genetic and pharmacological experiments reveal that cyclin E1-dependent signaling confers resistance to CDK4/6 inhibition whereas receptor tyrosine kinase (RTK) signaling contributes to CDK2 resistance. We further identify ETS transcription factors as critical downstream mediators of RTK signaling that are induced as part of the cell cycle machinery and cooperate with E2F transcription factors in controlling proliferation. Our results suggest that, due to the ability of cyclin D- and cyclin E-dependent signaling pathways to compensate for one another, in conjunction with frequent genetic alterations in HGSOC affecting both signaling arms, CDKi may not be efficient as single agents in the majority of HGSOC. Instead, our data indicate that CDKi may be most useful in combination therapy for genetically defined subsets of cancers. In a proof-of-principle study we show that dinaciclib can sensitize cyclin E1-dependent cells to platinum-based chemotherapy. In order to stratify patients for dinaciclib treatment, *CCNE1* amplification detectable by fluorescence *in situ* hybridization (FISH) or Southern Blot, is readily available as a companion diagnostic. Therefore, our study outlines a rational approach to incorporate CDKi into ovarian cancer treatment regimens.

## RESULTS

### CDKi impair E2F target gene expression and inhibit ETS gene transcription

**Figure 1:**
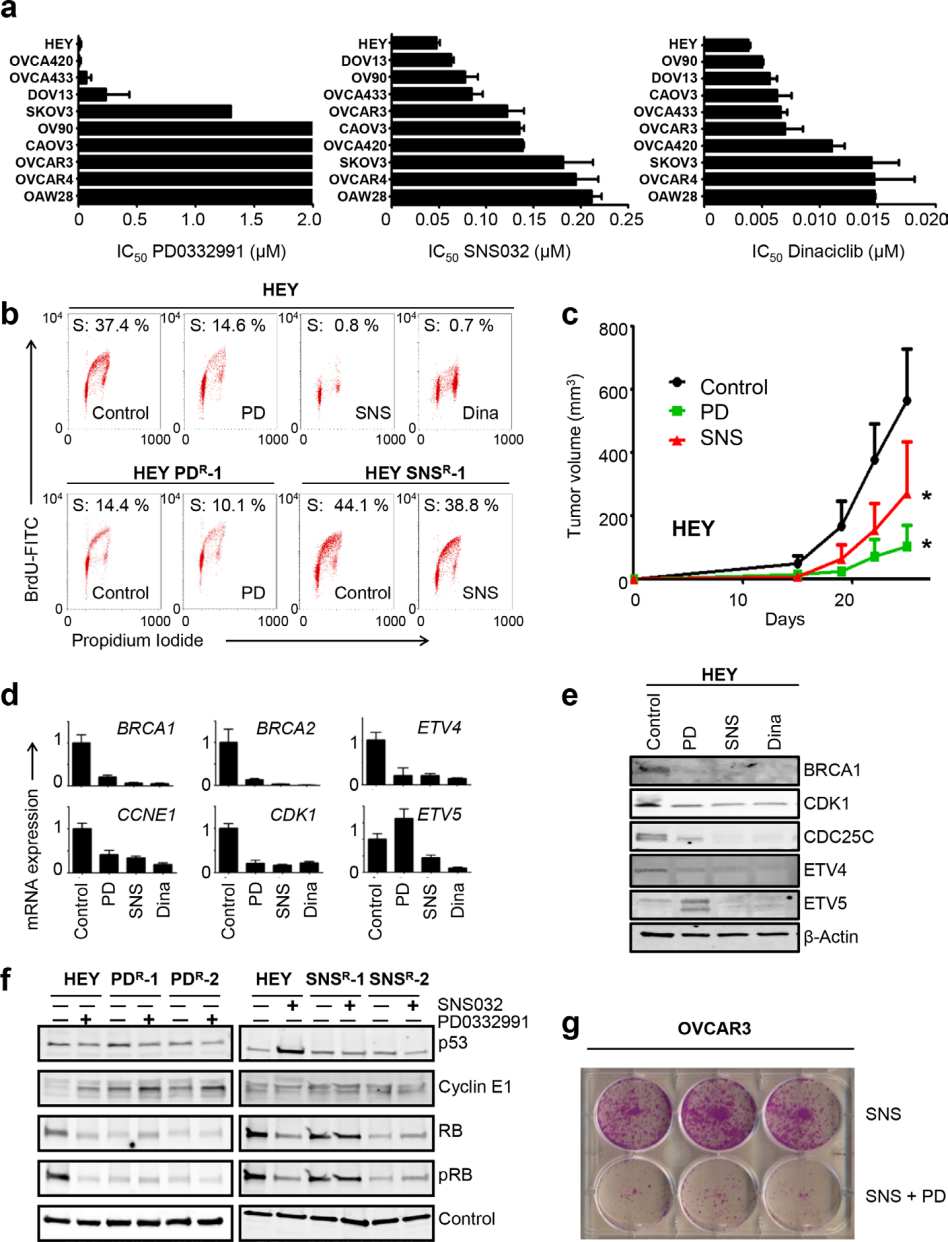
CDKi impair E2F target gene expression and inhibit ETS gene transcription **(a)** IC_50_ values for CDK4/6 inhibitor, PD0332991 (palbociclib) and CDK2 inhibitors, SNS032 and dinaciclib in 10 ovarian cancer cell lines. Cell viability was measured using a luminometric assay following 7d (PD0332991) or 72 h (SNS032, dinaciclib) of exposure to increasing concentrations of drug. The IC_50_ values displayed represent the mean plus SEM (*n* = 2) ranked from top to bottom from the most sensitive (HEY) to the most resistant (OAW28). PD0332991-resistant cell lines have IC_50_ values > 2 μM, while PD0332991-sensitive lines have IC_50_ values < 0.5 μM. SKOV3 cells display intermediate sensitivity. In contrast, IC_50_ values for SNS032 and dinaciclib fall along a continuum (0.05–0.25 μM for SNS032, 0.005–0.015 μM for dinaciclib). **(b)** CDKi-resistant cells avoid CDKi-mediated cell cycle arrest. Naïve HEY cells were treated with vehicle (control), 0.5 μM PD0332991, 0.2 μM SNS032 or 0.02 μM dinaciclib for 48 h and analyzed by BrdU/PI staining. HEY-PD^R^ and HEY-SNS^R^ cells were treated with 0.5 μM PD0332991 and 0.2 μM SNS032, respectively for 48 h. A representative FACS image is shown for each condition. **(c)** PD0332991 and SNS032 delay progression of HEY xenografts. HEY cells were injected into nude mice and treated with vehicle (control), 50 mg/kg PD0332991 or 15mg/kg SNS032 on day 8, 12, 15, 19 and 22 after injection. The data shown represent the mean tumor volume plus SEM (*n* = 6). *p = 0.04 (SNS032-treated versus control) and *p* = 0.0002 (PD0332991-treated versus control). **(d), (e)** Downregulation of E2F target genes and ETS factors by CDKi. HEY cells were treated with vehicle (control), 0.5 μM PD0332991, 0.2 μM SNS032 or 0.02 μM dinaciclib for 48 h with subsequent RNA and protein isolation. Validation of candidate target genes by (d) qPCR and (e) Western blot analysis are shown. **(f)** Biochemical changes in CDKi-resistant cells. Lysates derived from two HEY-PD^R^ clones (left) and two HEY-SNS^R^ clones (right) were treated in the absence or presence of 0.2 μM SNS032 or 0.5 μM PD0332991, respectively, for 48 h and probed with the antibodies indicated. Naïve HEY cells were included as controls. SNS032 induces TP53 protein in naïve HEY but not in HEY-SNS^R^. Elevated cyclin E1 and reduced RB protein levels are present in HEY-PD^R^. **(g)** CDK2 inhibition sensitizes OVCAR3 cells to PD0332991. OVCAR3 cells were treated with 0.2 μM SNS032 in the absence or presence of 0.5 μM PD0332991. Cell viability was determined by crystal violet staining after 3 months.

In order to assess the therapeutic potential of CDKi in HGSOC, we determined responses of ovarian cancer cell lines to three CDKi with different CDK specificity and selectivity: PD0332991 (palbociclib), SNS032 and dinaciclib (Fig. 1a, Supplementary Table 1). Previous studies have established *RB1* proficiency and *CDKN2A* (p16^INK4A^) deletion as the main determinants of PD0332991 sensitivity [9, 10]. Using a luminometric viability assay, we tested PD0332991 sensitivity in a panel of 10 ovarian cancer cell lines with different signature genetic alterations (Supplementary Table 2). We confirmed that *CDKN2A*-deficient cell lines were sensitive to CDK4/6 inhibition (IC_50_ values < 1 μM; HEY, DOV13, Fig. 1a). The SKOV3 cell line, while *CDKN2A*-null, showed intermediate sensitivity (Fig. 1a). Biochemically, PD0332991-sensitive cell lines (OVCA420, OVCA433, HEY, DOV13) had low expression of cyclin E1 RNA and protein with concomitant high expression of RB protein (Supplementary Fig. 1a). In contrast, cell lines with high cyclin E1 expression and low RB (CAOV3, OVCAR3, OVCAR4, OAW28, all with genomic *RB1* loss and/or *CCNE1* gain, Supplementary Table 2) were resistant to PD0332991 (Fig. 1a).

Next, we determined IC_50_ values for the CDK2 inhibitors SNS032 and dinaciclib. OAW28, OVCAR4 and SKOV3 were most resistant to both drugs (Fig. 1a, SNS032 IC_50_ ≥ 181.6 nM, dinaciclib IC_50_ ≥ 14.5 nM), whereas HEY, DOV13 and OV90 were most sensitive (Fig. 1a, SNS032 IC_50_ ≤ 77.7 nM, dinaciclib IC_50_ ≤ 5.7 nM). Two of the three most sensitive cell lines (HEY, DOV13) were *TP53*-wildtype and expressed low levels of cyclin E1 RNA and protein. In contrast, two of the three most resistant lines (OAW28, OVCAR4) were *TP53*-mutant and expressed high levels of cyclin E1 (Supplemental Fig. 1a, Supplementary Table 2). OVCAR3, the only *CCNE1*-amplified cell line in this panel, displayed intermediate sensitivity for SNS032 and dinaciclib (Fig. 1a, 122.2 nM and 7.0 nM, respectively). We conclude that additional factors besides cyclin E1 expression level determine cellular response to CDK2 inhibition. Similarly, reduced cell viability following cyclin E1 depletion was not limited to cyclin E1-overexpressing lines (Supplementary Fig. 1b). This suggests that the majority of ovarian cancer cell lines are cyclin E1/CDK2-dependent, confirming earlier reports [15]. Mechanistically, we found that PD0332991 induced a strong G_1_ arrest in sensitive cell lines, as reported before (Supplementary Fig. 1c) [9, 10]. In contrast, SNS032 and dinaciclib induced a mixed response in most cell lines that included G_1_ and G_2_/M arrest, as well as apoptosis (Supplemental Fig. 1c). Thus, whereas ovarian cancer cell lines can be clearly segregated into PD0332991-sensitive and resistant lines, IC_50_ values for current CDK2 inhibitors show a continuum within a narrow dose range that may be the result of non-cell cycle CDKs affected by SNS032 (CDK9) and dinaciclib (CDK5, 9). In order to study the effects of CDK2 inhibition on the cell cycle we therefore chose drug concentrations that induced cell cycle arrest but no apoptosis in the most resistant cell lines within 48 h (0.2 μM SNS032 and 0.02 μM dinaciclib).

Next, we compared the molecular effects of PD0332991 and SNS032 in the cell line HEY (*RB1* wildtype, *CCNE1* wildtype) since it was the most sensitive cell line in our panel for all three CDKi (Fig. 1a). Further validation of the efficacy of CDKi in this cell line was obtained by flow cytometry: Exposure of HEY cells to SNS032 or PD0332991 for 48 h drastically reduced the number of cells in S phase, as determined by BrdU incorporation assay (Fig. 1b, upper). Moreover, in subcutaneous HEY xenografts, treatment with SNS032 or PD0332991 delayed tumor progression compared to controls (Fig. 1c). Expression profiling followed by Ingenuity Pathway Analysis (IPA) established that the cell cycle was the most significantly affected cellular function in CDKi-treated cells (Supplemental Fig. 1d). IPA predicted E2F4, TP53 and E2F1 as the top regulators affected by PD0332991, and E2F4, ERBB2 and TP53 as the top regulators affected by SNS032 (Supplemental Fig. 1d). Established E2F target genes, such as *CDK1*, *CDC25C*, and *BRCA1*, were significantly downregulated in CDKi-treated cells (Table 1). Using RT-qPCR (qPCR), we validated downregulation of *BRCA1*, *BRCA2*, *CDK1* and *CCNE1* in response to PD0332991, SNS032, and dinaciclib (Fig. 1d). Downregulation of *BRCA1* and *CDK1* was further confirmed by Western blot (Fig. 1e). In addition to known E2F target genes, SNS032 caused downregulation of ETS family transcription factors, including *ELF2, ETV4* and *ETV5* (Table 1, Fig. 1d, e). Thus, we confirmed E2F-responsive genes as common targets of different CDKi and identified several ETS family members as potential cell cycle regulators affected by CDK2 inhibition.

**Table 1:** CDKi-resistant cells restore expression of E2F target genes and ETS family genes Relative expression of known E2F targets (^a^ from Bracken *et al*., 2004 [40]) and other select genes following exposure to 0.5 μM PD0332991 or 0.2 μM SNS032 in HEY cells for 48 h (left). Relative expression comparing HEY-SNS^R^ to SNS-exposed HEY cells and HEY-PD^R^ to PD0332991-exposed HEY cells (right). NS, not significant.

Symbol	Fold Change Parental vs PD-treated	*P*-value	Fold Change PD^R^ vs PD-treated	*P*-value
**Known E2F target genes ^a^**				
*CCNA1*	−4.24	0.01	1.75	0.03
*CCNE1*	NS		3.08	9.50E-03
*CDC6*	−2.39	7.04E-03	−1.68	5.57E-03
*CDK1*	−3.14	0.02	1.82	0.04
*CENPE*	−2.01	4.16E-03	2.26	1.35E-03
*RB1*	NS		−1.61	0.05
**Additional genes**				
*CCND2*	NS		8.26	5.60E-03
**Symbol**	**Fold Change Parental vs SNS-treated**	***P*-value**	**Fold Change SNS^R^ vs SNS-treated**	***P*-value**
**Known E2F target genes^a^**				
*AURKB*	−13.54	0.04	9.37	4.81E-03
*BUB1*	−15.75	1.73E-03	10.63	6.37E-04
*CCNA2*	−15.91	0.02	10.93	0.02
*CDC6*	−5.24	8.31E-03	5.61	4.56E-03
*CENPE*	−30.21	9.21E-04	5.19	0.05
*MKI67*	−15.58	4.36E-03	9.92	0.03
*MYB*	−6.37	0.04	10.32	0.03
*MYC*	−3.2	0.05	3.89	0.03
*NDC80*	−17.13	7.32E-05	11.38	0.04
*TTK*	−44.33	1.01E-05	29.54	0.02
**Additional genes**				
*CCNB1*	−23.55	0.01	20.01	0.02
*CDC25B*	−23.88	5.54E-04	16.59	7.12E-03
*CDC25C*	−34.96	8.09E-04	21.97	0.02
*CDKN1A*	64.18	0.03	−48.19	0.03
*ELF2*	−33.7	0.04	27.65	5.76E-03
*ETV4*	−2.74	0.02	2.23	0.03
*ETV5*	−3.49	7.43E-03	3.31	0.01
*EZH2*	−5.91	4.96E-03	5.92	4.15E-03
*FOS*	14.92	0.03	−13.79	0.04
*FOSB*	14.61	0.03	−17.94	0.03
*ID2*	63.52	0.03	−80.6	0.03
*JUNB*	21.27	0.03	−11.04	0.04
*PIK3CB*	−12.86	1.18E-03	12.66	7.28E-03

### CDKi-resistant ovarian cancer cell lines restore E2F and ETS function

To address if regained proliferative potential in CDKi-resistant cells involves restoration of E2F and ETS function, we assessed gene expression in CDKi-resistant HEY cells (Table 1, Supplementary Fig. 1d, 2a). First, we generated CDKi-resistant cells by isolating colonies after several months of selection in the presence of either 0.5 μM PD0332991 or 0.2 μM SNS032. For SNS032 selection, resistant colonies originated from single, surviving cells in low-density seeding conditions (500,000 cells/10 cm plate). For PD0332991 selection, cells were repeatedly passaged at low density until the onset of senescence in the majority of cells (Supplementary Fig. 2b). We previously established senescence as an endpoint of long-term PD0332991 exposure [10]. Resistant colonies that escaped from senescence were expanded in the presence of PD0332991 for at least ten passages prior to analysis. Next, we tested responses of resistant cells to other CDKi: HEY-SNS^R^ sustained proliferation in the presence of SNS032 (Fig. 1b, lower, Supplementary Fig. 2a) but remained sensitive to PD0332991 (Supplementary Fig. 2a, c). HEY-SNS^R^ cells were also less sensitive to dinaciclib (Supplementary Fig. 2a). In contrast, HEY-PD^R^ cells conferred resistance to PD0332991 but remained sensitive to SNS032 and dinaciclib (Supplementary Fig. 2a). These results suggested different adaptive responses in HEY-PD^R^ and HEY-SNS^R^.

In order to identify the molecular differences in adaptation we compared gene expression profiles for HEY-PD^R^, HEY-SNS^R^ cells, untreated HEY cells and CDKi-treated HEY cells. HEY-SNS^R^ cells had completely restored expression of most E2F target genes and clustered with naïve HEY cells (Table 1, Supplementary Fig. 1d). Similarly, expression of ETS family genes was comparable to that in parental cells (Table 1, right). In contrast, PD0332991-resistant HEY cells (HEY-PD^R^) did not regain their full proliferative potential in the presence of PD0332991 (Fig. 1b, lower) and did not fully restore E2F target gene expression (Table 1, right). On the level of individual genes, we compared PD0332991-treated and PD0332991-resistant cells and found that *CCNE1* was significantly upregulated and *RB1* was significantly downregulated in HEY-PD^R^ (Table 1, right) even though expression of both genes was not significantly affected by PD0332991 treatment of naïve HEY cells (NS in Table 1, left). These data suggest that the observed changes were part of the adaptive response in HEY-PD^R^ cells.

Indeed, biochemical analysis of protein expression in HEY-SNS^R^ and HEY-PD^R^ cells confirmed higher levels of cyclin E1 and lower levels of RB protein in HEY-PD^R^ cells (Fig. 1f). In HEY-SNS^R^ cells, cyclin E1 was not affected and RB was downregulated in only one clone (Fig. 1f). In contrast, HEY-SNS^R^ cells, unlike naïve HEY cells, avoided p53 protein accumulation in response to SNS032 exposure (Fig. 1f, Supplementary Fig. 2d). Thus, HEY-PD^R^ and HEY-SNS^R^ cells acquire different molecular changes. Further, long term exposure to PD0332991 and SNS032 in combination prevented the outgrowth of resistant cells in both HEY and OVCAR3 cells more effectively than single drug treatment (Fig. 1g, Supplemental Fig. 2e) but did not enhance the acute cytotoxic response elicited by SNS032 (Supplementary Fig. 2f). Combined treatment with dinaciclib and PD0332991 had a similar effect in OVCAR3 cells (Supplementary Fig. 2e). Thus, we conclude that resistance to PD0332991 and SNS032 involves different mechanisms that independently restore E2F target gene expression.

### *De novo* copy number aberrations are associated with restored E2F and ETS function

**Figure 2:**
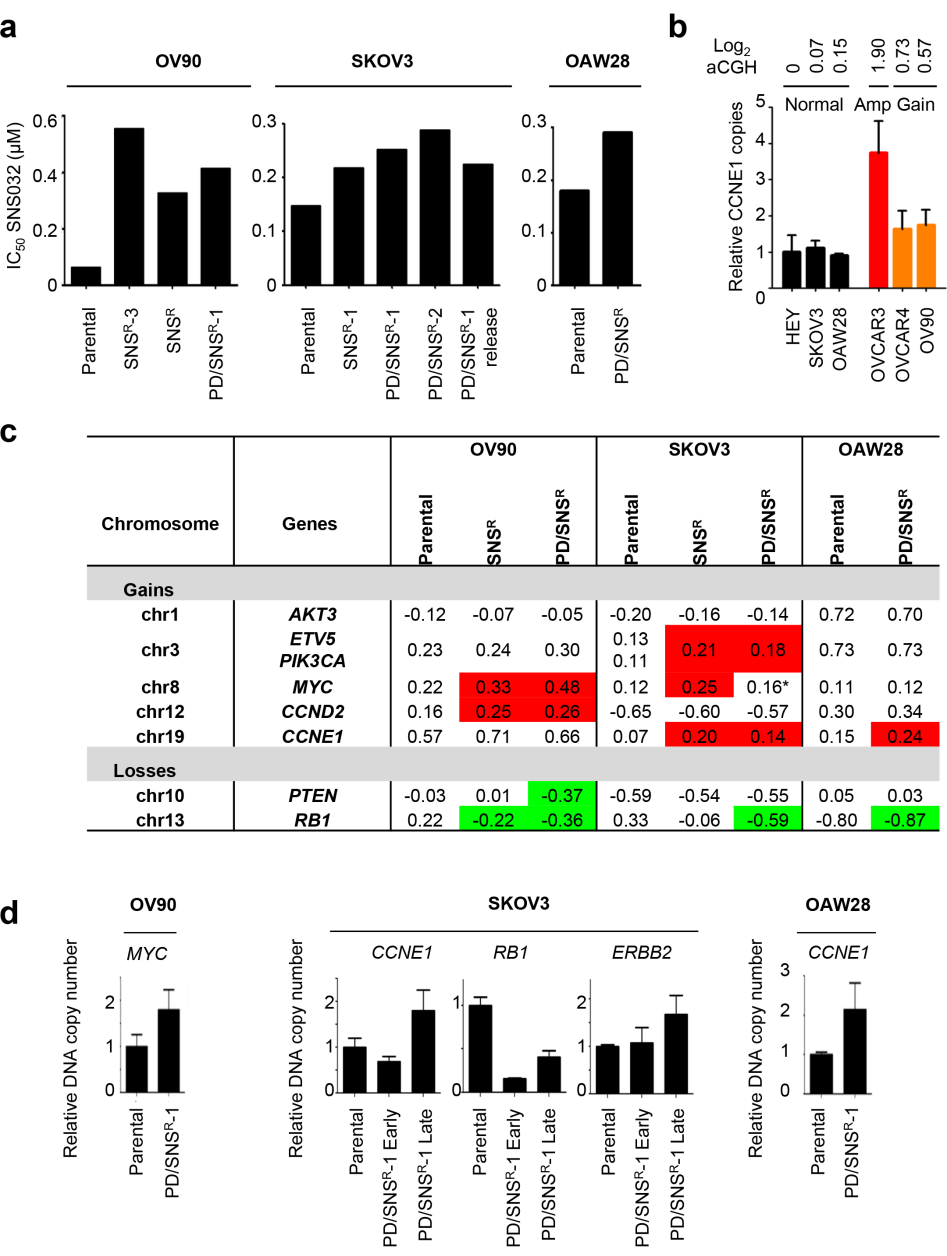
*De novo* copy number alterations in CDKi-resistant ovarian cancer cell lines **(a)** CDKi-resistant OV90, SKOV3 and OAW28 cells were generated by chronic exposure of parental cells to either 0.2 μM SNS032 alone or a combination of 0.2 μM SNS032 and 0.2 μM PD0332991. IC_50_ values were determined using a luminometric viability assay. **(b)** Relative *CCNE1* copy number across six ovarian cancer cell lines as determined by genomic qPCR and aCGH. **(c)** Recurrent gains and losses associated with CDKi resistance. Genomic DNA was isolated from parental and polyclonal populations of CDKi-resistant cells and subjected to aCGH analysis. Segmented log_2_ values are shown for select genes. Red indicates *de novo* copy number gain, green indicates *de novo* copy number loss. *a focal amplification region close to the *MYC* locus is present in these cells. **(d)** Validation of aCGH results by genomic qPCR. Genomic DNA was isolated from OV90, SKOV3 and OAW28 parental and CDKi^R^ cells. Relative DNA copy number for *CCNE1*, *RB1*, *ERBB2* and *MYC* was determined by qPCR.

Next, we asked if we could find genetic evidence for *de novo* alterations associated with CDKi resistance. To this end, we generated CDKi-resistant cell clones from three additional ovarian cancer cell lines. Since virtually all HGSOC harbor *TP53* mutations [2] we chose *TP53*-mutant OV90 and OAW28, as well as *TP53*-null SKOV3 (Supplemental Table 2). We reasoned that combined selection with PD0332991 and SNS032 would be an effective tool to study acquired resistance mechanisms affecting the cell cycle rather than apoptosis because (1) in contrast to SNS032, PD0332991 has no cytotoxic effect even at low micromolar concentrations and may push exposed cells towards arrest rather than death (Supplementary Fig. 1c), and (2) the addition of PD0332991 was able to enhance long-term SNS032 efficacy even in cell lines that are inherently PD03320991-resistant (e.g. OVCAR3, Fig. 1g). Thus, we generated both SNS032-resistant and PD0332991/SNS032-resistant sublines by selection in 0.2 μM SNS032 +/− 0.2 μM PD0332991 for 6 months (OV90, OAW28) or 1 year (SKOV3), resulting in polyclonal and monoclonal CDKi-resistant sublines (Fig. 2a). The resulting cell lines were analyzed by array-based comparative genomic hybridization (aCGH, Supplementary Table 3) and genomic qPCR (Fig. 2b) to detect DNA copy number changes. As predicted by our results in HEY cells, we found genomic *RB1* loss and *CCNE1* gain in PD0332991-selected cells (Fig. 2c, d). *De novo CCNE1* gain resulted in increased cyclin E1 protein expression in CDKi-resistant OAW28 cells (Supplementary Fig. 3a).

In contrast, SNS032 resistance was characterized by *de novo* aberrations in the receptor tyrosine kinase (RTK) signaling pathway (*ERBB2*, *AKT3*, and *PIK3CA* gain) as well as gain of *MYC* and *CCND2* (Fig. 2c, d). Activation of RTK signaling was demonstrated by increased ERBB2 expression in CDKi-resistant OAW28 and OV90 (Supplementary Fig. 3a). Expression profiling confirmed that these *de novo* aberrations were associated with restored E2F-mediated transcription in CDKi-resistant cells (Supplementary Table 4). In order to validate these findings in an independent system we analyzed three dinaciclib-resistant subclones of the OVCAR5 cell line for changes in ERBB2 and cyclin E1 expression. Dinaciclib-resistant OVCAR5 had considerably higher IC_50_ values for both dinaciclib (> 0.02 μM versus < 0.01 μM in parental) and SNS032 (> 0.1 μM versus 0.05 μM in parental) than parental OVCAR5 (Supplementary Fig. 3b). Increased expression of both ERBB2 and cyclin E1 was found in all three clones relative to parental controls (Supplementary Fig. 3b). Thus, multiple concomitant *de novo* aberrations drive CDKi resistance, including loss of *RB1*, gain of cyclins *CCNE1* and *CCND2*, and activation of RTK and MYC signaling.

### Cyclin E1 and ERBB2 confer resistance to CDKi

**Figure 3:**
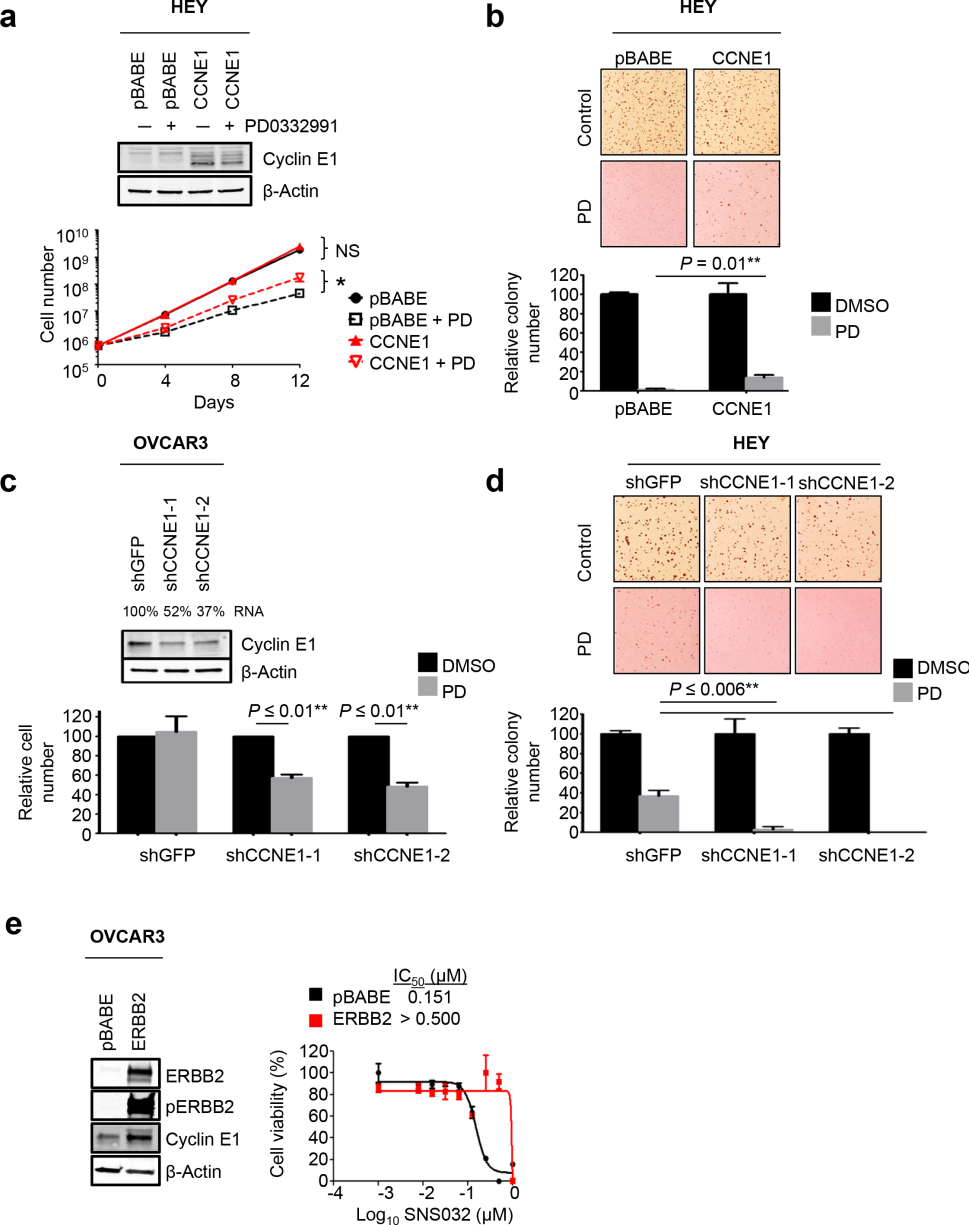
Cyclin E1 and ERBB2 confer resistance to CDKi **(a)** HEY cells were stably transduced with retroviral pBABE-CCNE1 (red lines) or empty vector (black lines). Stable polyclonal populations were treated with 0.5 μM PD0332991 or vehicle over a course of 12 days and counted every four days. Ectopic cyclin E1 expression was confirmed by Western blot analysis and had no effect on proliferation in regular culture conditions (solid lines) but conferred partial resistance in the presence of PD0332991 (dotted lines). *p = 0.002 **(b)** Ectopic cyclin E1 expression rescues soft agar colony formation in PD0332991-treated HEY cells. Cells were seeded into soft agar followed by treatment with 0.2 μM PD0332991 or vehicle. Colonies were stained and counted after 2 weeks. Columns represent the relative mean plus SEM (*n* = 3). *p = 0.01 **(c)** Depletion of cyclin E1 sensitizes OVCAR3 and **(d)** HEY cells to PD0332991-induced proliferation arrest. Two individual lentiviral shRNAs targeting *CCNE1* were used to genetically deplete cyclin E1 in OVCAR3 and HEY cells. A shRNA targeting GFP was used as a control. After puromycin selection, cells were harvested to confirm *CCNE1* knockdown by qPCR (relative *CCNE1* expression is given as a percentage) and Western blot analysis (c, upper). Cells were seeded for viability using a luminometric assay in the absence or presence of 0.5 μM PD0332991 for 6d. Columns represent the relative mean plus SEM (*n* = 3) *p ≤ 0.01 (c, lower). HEY-shGFP and HEY-shCCNE1 cells were seeded into soft agar and treated with vehicle or 0.2 μM PD0332991 (d, upper). Bar graph represents relative colony number compared to vehicle plus SD (*n* = 3). *p ≤ 0.006. **(e)** Ectopic expression of mutant ERBB2 confers resistance to SNS032. OVCAR3 cells were stably transduced with retroviral pBABE-ERBB2 or empty vector. Stable polyclonal populations were treated with increasing concentrations of SNS032, and viability was assessed by a luminometric viability assay.

While *RB1* deletion is a well-established mechanism of resistance to PD0332991 [8–10], the role of cyclin E1 and ERBB2 in CDKi resistance has not been established yet. Concomitant activation of ERBB2 and cyclin E1 in both PD0332991/SNS032-selected and dinaciclib-selected cells suggested that both drivers contribute to CDKi resistance. We next designed functional experiments to test the individual impact of each gene on CDKi resistance. To study cyclin E1 in gain-of-function experiments, we chose PD0332991-sensitive HEY cells with low endogenous cyclin E1 expression. HEY cells were engineered to constitutively express a cyclin E1 transgene following retroviral transduction (HEY-CCNE1). Cells transduced with an empty retroviral construct were used as controls (HEY-pBABE). In luminometric viability assays, HEY-CCNE1 cells displayed significantly increased resistance to PD0332991 (Supplementary Fig. 2a, pBABE versus CCNE1-1 and CCNE1-2, *p* = 0.03 and *p* = 0.01, respectively) but not SNS032 or dinaciclib (Supplementary Fig. 2a). We then compared HEY-pBABE and HEY-CCNE1 cells in proliferation assays (Fig. 3a) and in soft agar colony formation assays, a surrogate of anchorage-independent growth (Fig. 3b). Ectopic cyclin E1 expression had no proliferation-enhancing effect under regular culture conditions but conferred a significant proliferation advantage in the presence of PD0332991 (Fig. 3a, *p* = 0.002). Similar results were obtained for another PD0332991-sensitive ovarian cancer cell line with low endogenous cyclin E1 expression, OVCA433 (Supplementary Fig. 3c). These data indicate that overexpression of cyclin E1, while unable to confer complete PD0332991 resistance in the absence of additional factors (e.g. *RB1* loss), is sufficient to rescue PD0332991-induced proliferation arrest. Indeed, in long-term colony formation assays, HEY-pBABE cells underwent senescence within four weeks of treatment under low density seeding conditions, similar to naïve HEY cells (Supplemental Fig. 2b). In contrast, HEY-CCNE1 cells and *CCNE1*-amplified OVCAR3 cells were resistant to PD0332991-induced senescence (Supplemental Fig. 2b). In soft agar PD0332991 efficiently suppressed colony formation in HEY-pBABE cells, whereas ectopic cyclin E1 partially rescued PD0332991-mediated suppression of colony formation (Fig. 3b, *p* = 0.01). Taken together, these observations suggest that overexpression of cyclin E1 as a result of ectopic expression or amplification is sufficient to render ovarian cancer cells resistant to PD0332991-induced proliferation arrest.

Next, we investigated if cyclin E1 is required for PD0332991 resistance. In PD0332991-resistant OVCAR3 (*RB1* loss, *CCNE1* amplification) cells, we used two individual lentiviral shRNAs to genetically deplete cyclin E1. Reduction of cyclin E1 RNA levels by 50 – 70% (Fig. 3c) sensitized OVCAR3 cells to PD0332991, as determined by luminometric viability assays (Fig. 3c, *p* ≤ 0.01). We performed similar experiments in cyclin E1-depleted HEY and DOV13 cells (Supplemental Fig. 3d). Cyclin E1 depletion further increased PD0332991 susceptibility in both cell lines and led to a near-complete loss of anchorage-independent growth in the presence of PD0332991 in HEY cells (Fig. 3d, *p* ≤ 0.006). These results suggested that cyclin E1 is required for PD0332991 resistance in ovarian cancer cells and that inhibition of cyclin E1 signaling can improve response to PD0332991. We did not test any *RB1*-null cell lines in this study but speculate that cyclin E1 may be obsolete for PD0332991 resistance in an *RB1*-null setting. In summary, cyclin E1 signaling confers resistance to PD0332991 in *RB1*-proficient ovarian cancer cells.

Since activated RTK and PI3K signaling was a common motif in CDK2 inhibitor-resistant ovarian cancer cells, we next tested the hypothesis that constitutively active RTK signaling could confer resistance to CDK2 inhibition. To this end, we introduced a mutant form of ERBB2 into *CCNE1*-amplified OVCAR3 cells (Fig. 3e). Overexpression of mutant ERBB2 dramatically increased the IC_50_ value for SNS032 from 150.7 nM to up to 500 nM (Fig. 3e). Moreover, OVCAR3-ERBB2^mut^ cells were also more resistant to dinaciclib (Supplementary Fig. 3e). Thus, increased RTK signaling can protect *TP53*-deficient cells with high cyclin E1 expression against CDK2 inhibition.

### ETS transcription factors are downstream mediators of RTK/RAS signaling

**Figure 4:**
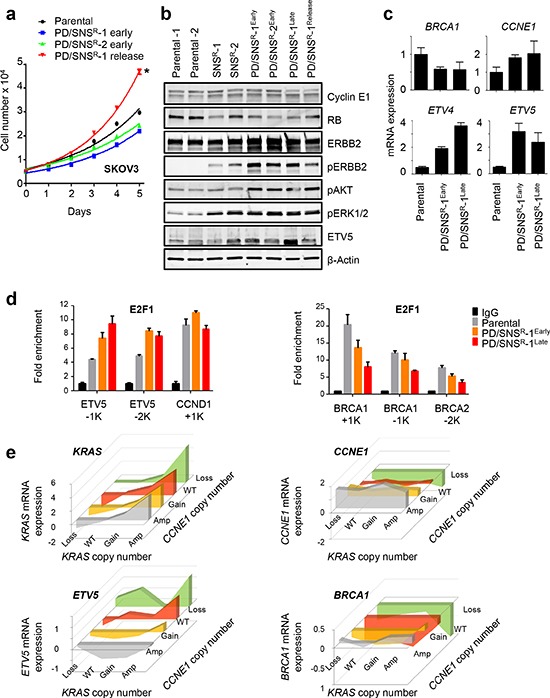
ETV5 is a downstream mediator of RTK/RAS signaling **(a)** SKOV3-PD/SNS^R^ release cells are hyperproliferative. CDKi-resistant SKOV3 cells were generated by chronic exposure to either 0.2 μM SNS032 alone or a combination of 0.2 μM PD0332991 and 0.2μM SNS032 for up to two years. SKOV3-PD/SNS^R^ release cells were analyzed after 6 months in the absence of CDKi following two years of exposure. Proliferation rates of SKOV3-PD/SNS^R^, release and parental cells were determined using a luminometric viability assay. *p = 5.36E-06. **(b)** Western blot analysis of CDKi-resistant SKOV3 cells using antibodies against RB, RTK and PI3K signaling markers. CDKi-resistant cells hyperactivate ERBB2 and its downstream effectors MAPK and AKT, resulting in increased ETV5 protein expression. **(c)** *ETV4* and *ETV5* are upregulated in CDKi-resistant SKOV3 cells. Gene expression in SKOV3-PD/SNS^R^ and parental SKOV3 cells was determined by qPCR. **(d)** Increased binding of E2F1 at the *ETV5* promoter in CDKi-resistant cells. Chromatin from parental and CDKi-resistant SKOV3 cells was isolated and used in a ChIP assay with an E2F1 antibody or IgG control. Relative enrichment (E2F1/IgG) at the *ETV5, CCND1* (left) and *BRCA1*, *BRCA2* (right) promoters is shown for naïve, early and late SKOV3-PD/SNS^R^ cells. **(e)** *ETV5* expression correlates with *KRAS* and *CCNE1* DNA copy number in primary HGSOC. Using the TCGA dataset, expression of *KRAS*, *ETV5*, *CCNE1* and *BRCA1* (y-axis) were plotted relative to *KRAS* (x-axis) and *CCNE1* (z-axis) copy number status. *ETV5* expression is positively correlated with *KRAS* copy number and inversely correlated with *CCNE1* copy number.

In order to identify the molecular mechanisms downstream of RTK signaling that mediate CDKi resistance we used the SKOV3 cell line. Since SKOV3 shows intermediate sensitivity to PD0332991 (Fig. 1a), selection with both SNS032 and PD0332991 resulted in a prolonged state of impaired proliferation similar to what we observed in HEY-PD^R^ cells (Fig. 1b). This allowed us to monitor emerging resistance over time (Supplemental Fig. 4a). We characterized both RNA and protein expression of several CDKi-resistant cell clones over a course of two years, comparing parental SKOV3 to SKOV3-SNS^R^-1 and 2 (profiled after one year), SKOV3-PD/SNS^R^-1 (profiled after 1 year of CDKi exposure (early), 2 years of CDKi exposure (late) and 6 months of release following 2 years of selection (release)) and SKOV3-PD/SNS^R^-2 (profiled after 1 year of CDKi exposure). SKOV3-PD/SNS^R^-early cells exhibited reduced expression of some E2F target genes (Supplementary Table 5), as well as reduced proliferation rates (Fig. 4a) despite loss of *RB1* (Fig. 2d). After another year in selection, SKOV3-PD/SNS^R^-late cells had increased expression of E2F target genes to levels that were comparable to or above those in parental cells (Supplementary Table 5). These expression changes coincided with copy number gains in *CCNE1* and *ERBB2* (Fig. 2d). Upon release from CDKi-induced suppression of proliferation for 6 months, SKOV3-PD/SNS^R^ cells acquired a hyperproliferative phenotype compared to parental SKOV3, suggesting that CDKi-associated *de novo* copy number aberrations (loss of *RB1*, gain of *CCNE1* and ERBB2) provided the cells with a proliferative advantage (Fig. 4a). Biochemically, ERBB2 signaling increased with added selection pressure (PD0332991/SNS032 versus SNS032 alone) and time, resulting in activation of MAPK and AKT signaling (Fig. 4b, Supplementary Fig. 4b, c). Hyperactivation of MAPK signaling was associated with increased expression of ETS factors *ETV4* and *ETV5* (Fig. 4b, c). *ETV5* maps to chromosome 3q27-28, which is frequently amplified in HGSOC and harbors a number of putative oncogenes including *MECOM*, *PIK3CA* and *TERC [2]*. The region gains additional copies in CDKi-resistant SKOV3 (Fig. 2c). However, copy number gains alone are insufficient to induce *ETV5* RNA as levels reverted back to baseline in SKOV3-PD/SNS^R^-1 release cells (Fig. 4c, Supplementary Fig. 4b). These results suggested a dynamic interaction between ETS and E2F transcription factors. Given that acute exposure to SNS032 resulted in downregulation of ETS factors, including *ETV4* and *ETV5* (Fig. 1d, e, Table 1), we next asked if E2F transcription factors are directly involved in the regulation of ETS genes, such as *ETV5*. Chromatin immunoprecipitation (ChIP) showed binding of E2F1 to proximal *ETV5* promoter elements that was more pronounced in CDKi-resistant SKOV3 than in parental cells and increased over time, with the highest binding observed in late SKOV3-PD/SNS^R^-1 cells (Fig. 4d). In contrast, binding of E2F1 to the *BRCA1* promoter, a classic E2F target, showed an inverse pattern, with the lowest binding observed in late SKOV3-PD/SNS^R^-1 cells (Fig. 4d). Collectively, these data show that E2F and ETS-mediated transcription is altered in CDKi-resistant ovarian cancer cells, reflecting the differential activation of different drivers of E2F-mediated transcription, including cyclin E1 and RTK/RAS signaling. We next asked if we could identify a similar pattern in primary HGSOC: Since *CCNE1* and *KRAS* are frequently amplified in HGSOC we plotted *ETV5* RNA expression as a function of *CCNE1* and *KRAS* DNA copy number, using publicly available TCGA data on 489 HGSOC tumors [2] that we accessed via the cBioPortal [22, 23]. *ETV5* expression was positively correlated with *KRAS* copy number and inversely correlated with *CCNE1* copy number (Fig. 4e). In contrast, *BRCA1* expression showed the opposite pattern with highest expression in *CCNE1*-amplified HGSOC. Thus, *ETV5* and *BRCA1* are E2F target genes that are associated with different drivers of E2F-mediated transcription (RTK/*KRAS*: ETV5; *CCNE1*: BRCA1).

Given that E2F link proliferation and DNA repair [24] and that BRCA1 is an important determinant of chemosensitivity in HGSOC [25] we then considered the possibility that CDKi may alter response to platinum-based drugs. Indeed, we found that sensitivity to cisplatin was frequently enhanced in CDKi-resistant cells (Supplementary Fig. 4d), coinciding with lower levels of *BRCA1* and *BRCA2* RNA (Fig. 4c, Supplementary Fig. 4e). Thus, disruption of E2F-mediated transcription can sensitize cells to platinum-based chemotherapy.

### ETV5 regulates cell cycle genes and contributes to tumorigenicity

**Figure 5:**
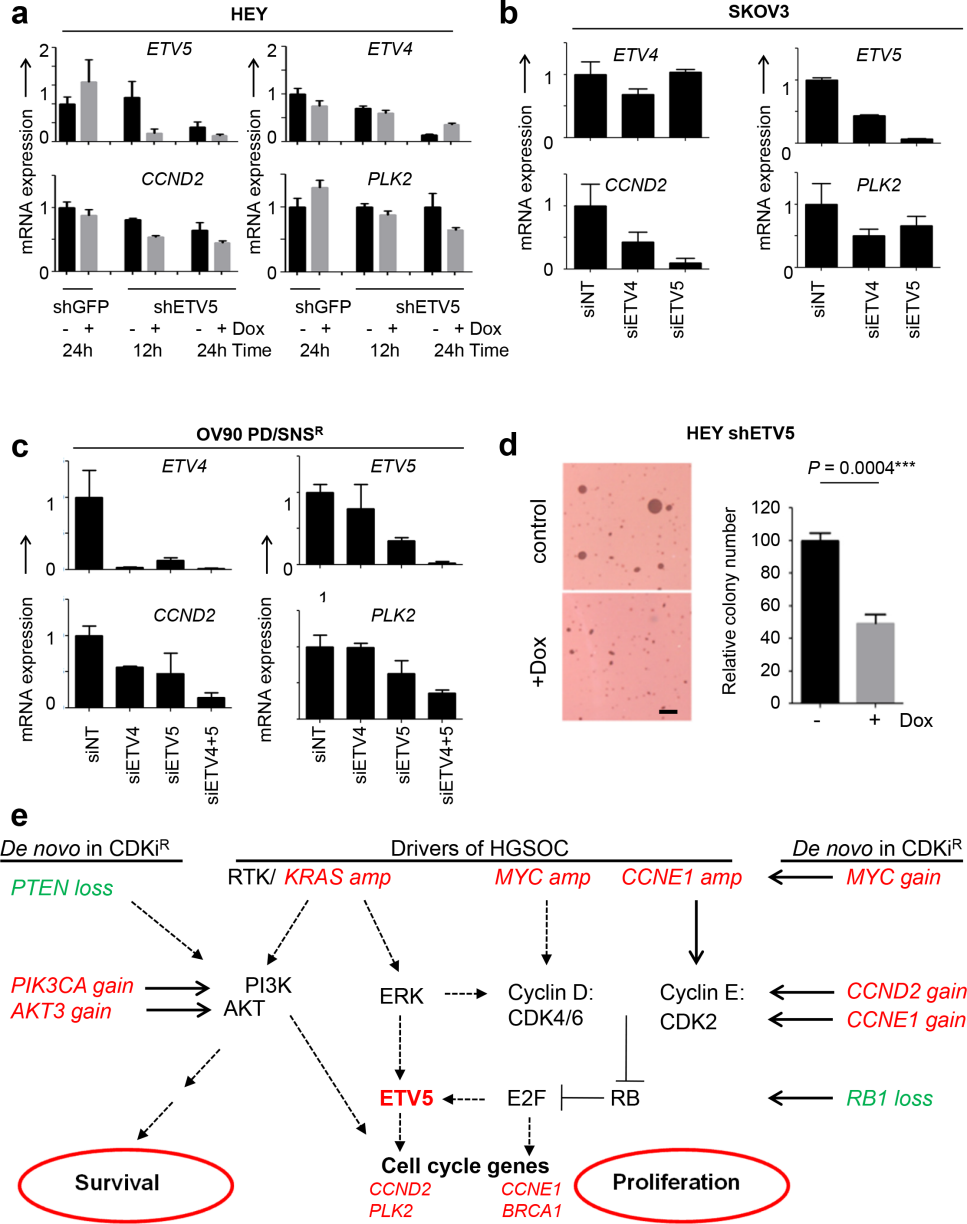
ETV5 controls cell cycle gene expression and contributes to tumorigenicity **(a)** ETV5 is required for expression of *CCND2* and *PLK2* in HEY cells. HEY cells with inducible knockdown of *ETV5* were generated by lentiviral infection with pTRIPZ-shETV5 followed by puromycin-selection. Doxycycline-induced ETV5 knockdown was compared with uninduced cells as well as shGFP control cells. Cells were grown in the absence (black bars) or presence (grey bars) of doxycycline for the indicated times, total RNA isolated and subsequently analyzed by qPCR. **(b)** Transient depletion of *ETV4* and *ETV5* in SKOV3 and **(c)** OV90-PD/SNS^R^ cells exhibited similar downregulation of ETS targets by qPCR as in a. **(d)** ETV5 is required for tumorigenicity. Inducible HEY-shETV5 cells were seeded into soft agar and treated with or without doxycycline for 24 h. Colonies were stained and counted after 2 wk (left). Columns represent the relative mean plus SEM (*n* = 3) (right). *p = 0.0004; Scale bar, 1 mm. **(e)** Model of cell cycle deregulation including drivers of HGSOC (in italics) and *de novo* copy number aberrations (gain, red; loss, green) found in CDKi-resistant ovarian cancer cell lines (in italics).

Mechanistically, these data suggested that *ETV5* expression is regulated by E2F. Since some E2F targets, such as *CCNE1*, also induce E2F activity as part of a positive feedback loop, we next asked if ETV5 contributes to cell cycle gene expression in a similar manner. In order to test if ETV5 is required for the expression of E2F targets in RAS-driven ovarian cancer cells we revisited the HEY cell line, which carries a KRAS^G12D^ mutation, as well as gain of the *KRAS* gene. We generated HEY subclones with inducible knockdown of ETV5 by lentiviral infection with pTRIPZ-shETV5 and compared gene expression in doxycycline-induced cells and uninduced cells, as well as HEY cells carrying a shGFP control hairpin (Fig. 5a). Genetic depletion of *ETV5* resulted in reduced expression of *CCND2* and *PLK2*, indicating that ETV5 is required for the expression of cell cycle genes (Fig. 5a). Transient depletion of ETV4 and ETV5 in *ERBB2*-amplified SKOV3 cells and OV90-PD/SNS^R^ cells, which have *CCND2* copy number gain (Fig. 2c), showed similar results (Fig. 5b, c). Soft agar colony formation assays further demonstrated that ETV5 is required for tumorigenicity in HEY cells. *ETV5* RNA depletion significantly reduced colony number (Fig. 5d, *p* = 0.0004). Collectively, our data support a model where E2F and ETS transcription factors cooperate to drive cell cycle gene expression (Fig. 5e): Drivers of HGSOC activate several signaling cascades that result in expression of cyclin D (downstream of KRAS-ERK signaling and a direct target of MYC) and/or cyclin E (e.g. as a result of *CCNE1* amplification) and subsequent activation of E2F target genes and proliferation. In parallel, ERK activates ETS transcription factors, including ETV5, which contribute to cell cycle progression via induction of cell cycle genes. Genetic changes in CDKi-resistant cells (gains in red, losses in green) affect multiple regulators of proliferation, which collectively restore cell cycle progression via E2F and ETS-mediated transcription. Resistance to CDKi that induce cell death (SNS032, dinaciclib) further requires the activation of survival pathways such as the AKT pathway.

### The CDKi, dinaciclib, sensitizes cyclin E1-driven ovarian cancers to cisplatin

**Figure 6:**
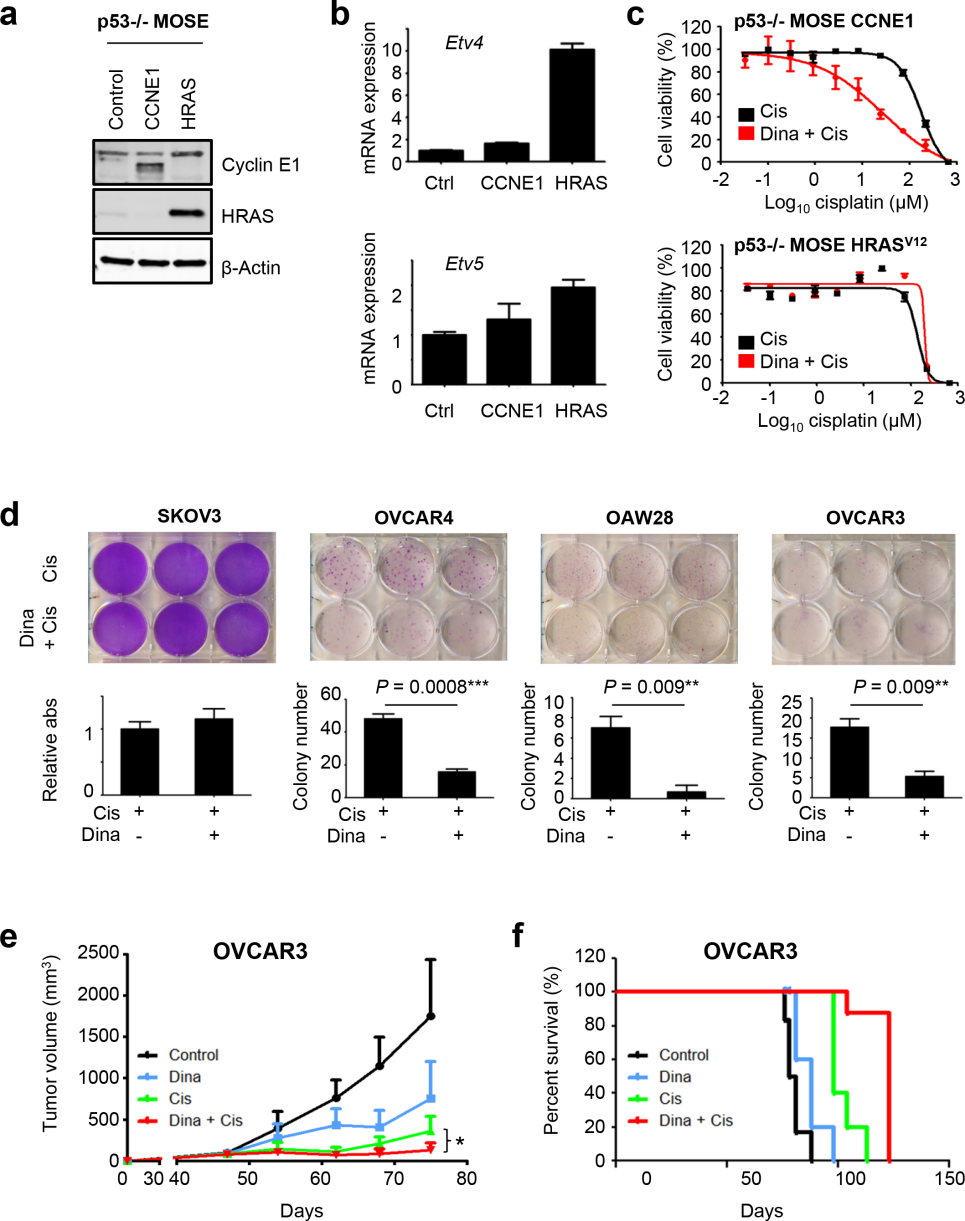
Dinaciclib sensitizes cyclin E1-driven ovarian cancers to cisplatin **(a)** p53−/− MOSE cells were transformed by a CCNE1 or HRAS^G12V^ transgene. Using the empty pBABE vector as a control, ectopic expression of cyclin E1 and HRAS was confirmed by Western blot analysis. **(b)** Overexpression of HRAS^G12V^ increases *Etv4* and *Etv5* expression. p53−/− MOSE-CCNE1 and p53−/− MOSE-HRAS^G12V^ cells were analyzed for *Etv4* and *Etv5* levels by qPCR. **(c)** Dinaciclib sensitizes p53−/− MOSE-CCNE1 cells but not p53−/− MOSE-HRAS cells to cisplatin. Cells were grown in the absence or presence of 0.03 μM dinaciclib for 24 h with increasing concentrations of cisplatin added for an additional 48 h. Cell viability was measured by a luminometric assay. **(d)** Dinaciclib enhances cisplatin efficacy in cyclin E1-dependent human ovarian cancer cells. Cells were treated with 2 μM cisplatin for two days followed by a two day treatment with 10 nM dinaciclib or vehicle. Following removal of drug, cells were cultured for an additional week prior to staining of surviving colonies by crystal violet. *p = 0.0008 (OVCAR4), *p* = 0.009 (OAW28, OVCAR3). **(e)** Combination treatment with dinaciclib and cisplatin significantly delay tumor growth and **(f)** survival. Established OVCAR3 xenografts were treated intraperitoneally with vehicle (control), 40 mg/kg dinaciclib alone, 2mg/kg cisplatin alone or a combination of 40mg/kg dinaciclib and 2 mg/kg cisplatin. Treatment was started on day 47 after injection, and continued once weekly over 4 weeks. The data shown represent the mean tumor volume plus SEM (*n* = 5) *p = 0.009 (e) and corresponding Kaplan-Meier survival curve (f) of the 4 treatment groups (*p* ≤ 0.0001).

Finally, we sought to test our model of differential cell cycle regulation in the context of ovarian cancer therapy. We hypothesized that in cyclin E1-driven cells, CDK2 inhibitors could be used as chemosensitizers based on their ability to downregulate *BRCA1* and other DNA repair genes that are critical determinants of cisplatin sensitivity (Fig. 1d, e, Supplemental Fig. 4d, e). As a genetically defined model of cyclin E1 dependence, we employed *Trp53*-null (p53 −/−) mouse ovarian surface epithelial (MOSE) cells [26] that were transformed by a *CCNE1* transgene (MOSE-CCNE1) or mutant *HRAS*^G12V^ (MOSE-HRAS). MOSE cells infected with empty pBABE vector were used as controls (Fig. 6a). We verified increased expression of *Etv4* and *Etv5* in MOSE-HRAS cells (Fig. 6b), suggesting that this model recapitulated the differences we observed in *CCNE1* and RTK/*RAS*-driven ovarian cancers. In MOSE-CCNE1 cells, dinaciclib had a dramatic effect on cisplatin sensitivity (Fig. 6c, Supplementary Fig. 5a), decreasing the IC_50_ from 165.0 μM to 32.9 μM. In contrast, MOSE-HRAS cells were not sensitized by dinaciclib. Indeed, drug combination studies confirmed that dinaciclib and cisplatin act synergistically in MOSE-CCNE1 cells but not in MOSE-HRAS cells (Supplementary Fig. 5b). Similarly, in human ovarian cancer cell lines with high endogenous cyclin E1, OVCAR3, OVCAR4 and OAW28 (Supplementary Fig. 1a), a single low dose of dinaciclib significantly reduced the number of cisplatin-surviving colonies (Fig. 6d, Supplementary Fig. 5c), whereas in SKOV3 cells with high endogenous ERBB2 and low cyclin E1 (Supplementary Fig. 1a), dinaciclib had no added effect on chemosensitivity. We then proceeded to test the combination *in vivo*: established OVCAR3 xenografts were randomized into four treatment groups and treated with vehicle, dinaciclib alone, cisplatin alone, or a combination of cisplatin and dinaciclib (Fig. 6e). The combination significantly delayed progression and increased survival compared to either single treatment group (Fig. 6e, f). In conclusion, dinaciclib can be a potent chemosensitizer in cyclin E1-dependent ovarian cancer cells.

## DISCUSSION

Signaling by cyclin-CDK complexes via RB and E2F activates E2F target gene expression, which can be inhibited by pharmacological CDK inhibitors. CDK inhibitors have entered late stage clinical trials for some human cancers but could have the potential for broader applicability due to near-universal genetic RB pathway aberrations found across many different cancer types [6]. However, mouse knockout models have elegantly demonstrated that genetic deletion of individual interphase CDKs has relatively little effect on organismal viability in general and cell cycle progression specifically [27–29]. For example, CDK2−/− mice are viable [30], and silencing of CDK2 had no significant impact on proliferation in some cancer cells [31]. In contrast, CDK2/4 double knockout results in embryonic lethality [32]. Using ovarian cancer as a model system, we have compared directly the acute effect of selective CDK4/6 inhibition (PD0332991 = palbociclib) or CDK2 inhibition (SNS032, dinaciclib), as well as mechanisms of resistance. Our data suggest that ovarian cancer cells adapt to CDK inhibition by acquiring, or selecting for, multiple *de novo* copy number aberrations that are upstream of cyclin D or cyclin E: Copy number gain and overexpression of *CCNE1*, as well as loss of *RB1*, was observed in PD0332991-resistant cells, and experimental modulation of cyclin E1 expression altered PD0332991 sensitivity, whereby overexpression of cyclin E1 conferred resistance to CDK4/6 inhibition, whereas genetic depletion of cyclin E1 increased sensitivity to CDK4/6 inhibition. In line with this, combined inhibition of CDK2/4/6 by PD0332991 and SNS032 prevented the formation of CDKi-resistant colonies *in vitro*. However, this combination may not be suitable *in vivo*, due to inherent functional antagonism and general toxicity: CDK4/6 inhibition results in G_1_ cell cycle arrest and is cytostatic in most systems whereas CDK1/2 inhibition leads to apoptosis, as shown by our data and published studies [33, 34]. These two opposing mechanisms may prevent effective tumor regression *in vivo* [35] and may explain the failure of earlier pan-CDKi, such as Flavopiridol, in the clinic.

Resistance to CDK2 inhibitors appears to be more complicated and involves multiple genetic changes, possibly because both SNS032 and dinaciclib inhibit other non-cell cycle CDK, such as CDK5 and CDK9, inducing both cell cycle arrest and apoptosis. Therefore, CDK2 inhibitor resistance requires the activation of survival pathways in addition to restoration of proliferative potential whereas PD0332991 resistance does not. A common theme observed in all CDK2 inhibitor-resistant cell lines was the selection for inducers of cyclin D signaling, such as *MYC*, *CCND2*, and RTK signaling. Within the RTK signaling network, we observed gain of *ERBB2*, *PIK3CA* and *AKT3*. In each CDKi-resistant cell line, several *de novo* changes in the pathway were observed, resulting in activation of both the MAPK signaling cascade, an inducer of proliferation via cyclin D, as well as AKT signaling, generally considered a survival pathway. In addition to cyclin D, MAPK signaling induces ETS transcription factors via activation of ERK, which results in transcriptional upregulation of ETS factors and post-translational modification of ETS proteins by phosphorylation (reviewed in [36]). We identified the ETS member ETV5 as a critical downstream effector of MAPK signaling in ovarian cancer cells. In MAPK-dependent ovarian cancer cells, ETV5 is both induced by E2F and required for the expression of cell cycle genes, such as *CCND2* and *PLK2*. Thus, genetic events, such as *CCND2* copy number gain, cooperate with increased ETV5 activity to drive expression of cyclin D2 when cyclin E-CDK2 activity is chronically inhibited.

Importantly, our findings are not limited to CDKi-resistant cell lines but apply to primary HGSOC. Analysis of TCGA data revealed that *ETV5* is highly expressed in *KRAS*-amplified HGSOC and low in *CCNE1*-amplified HGSOC, possibly reflecting a differential requirement of ETV5 in subgroups of HGSOC. Interestingly, *ETV5* is also frequently amplified in HGSOC as part of a large genomic region on chromosome 3q26-28 which also harbors *MECOM*, *PIK3CA*, and *TERC*. In CDK2 inhibitor-resistant cells, we found gain of this region, suggesting that both *PIK3CA* and *ETV5* may contribute to CDK2i resistance. Collectively, a picture emerges in which E2F target gene expression is controlled by multiple oncogenic pathways downstream of signature genomic aberrations of HGSOC, including *CCNE1* amplification, loss or deletion of *RB1*, as well as amplification of *MYC* and *KRAS*. The capacity of these pathways to functionally compensate for each other, in addition to the genomic complexity of HGSOC where multiple events among E2F regulators frequently occur (e.g. co-amplification of *CCNE1* and *KRAS*), will likely exacerbate the identification of responder populations for individual CDKi. Moreover, our data suggest that E2F and ETS transcription factors coordinately regulate cell cycle progression and cross-regulate each other. Similar to cyclin E1, ETV5 is both induced by E2F during cell cycle progression and required for the expression of a subset of cell cycle genes. The inverse correlation between *CCNE1* and *ETV5* expression may be indicative of a negative feedback mechanism - similar to E2F-mediated suppression of *CCND1* in late G_1_ - or may merely reflect the relative enrichment of different cell cycle phases in *CCNE1*-driven versus *KRAS*-driven tumors.

Given the vast potential for resistance as a result of genomic complexity is there a use for CDKi in the treatment of HGSOC? Due to the high prevalence of *CCNE1* gain/amplification and *RB1* loss, mutation or deletion, palbociclib is expected to be ineffective in the majority of primary HGSOC but may be selectively applied in cyclin E1-low, *RB1*-proficient cancers [9], or as follow-up treatment in CDK2 inhibitor-resistant cancers. Our data suggest that combination with RTK inhibitors or MAPK inhibitors may be effective, as shown in other cancers [37]. In contrast, CDK2 inhibitors, such as dinaciclib, may be most effective as chemosensitizers in cyclin E1-driven HGSOC, including *CCNE1*-amplified tumors. *CCNE1*-amplified HGSOC comprise 20% of all HGSOC and lack mutations in *BRCA1* and *BRCA2* [2]. *BRCA*-mutant cancers are associated with improved overall survival, in large part due to the increased platinum sensitivity as a result of compromised homologous recombination (HR)-mediated DNA repair function [38, 39]. *BRCA1* and *BRCA2* are also known E2F targets [40] and overexpressed in *CCNE1*-amplified HGSOC [25, 41] which require intact BRCA1 function [41]. Inhibition of CDK1/2 by SNS032 or dinaciclib resulted in downregulation of *BRCA1* and chemosensitization specifically in cyclin E1-driven cells. In summary, these findings may help to incorporate CDKi into rational therapeutic combinations in a subset of the most difficult to treat tumors.

## MATERIALS AND METHODS

### Cell culture and reagents

Human ovarian carcinoma cell lines CAOV3, OVCAR3, OV90 and SKOV3 were obtained from ATCC (Manassas, VA). OVCAR4 cells were purchased from NCI (Frederick, MD). OVCAR5, OAW28, HEY and DOV13 were kindly provided by Dr. D. J. Slamon (Los Angeles, CA). OVCA420 and OVCA433 were kindly provided by Dr. R. Drapkin (Boston, MA). Resistant cells were maintained in 0.5 μM PD0332991 (PD0332991-resistant, PD^R^), 0.2 μM SNS032 (SNS032-resistant, SNS^R^), 0.2 μM PD0332991/0.2μM SNS032 (PD0332991/SNS032-resistant, PD/SNS^R^) or 0.02 μM dinaciclib (dinaciclib-resistant, Dina^R^). STR profiling was used to authenticate each cell line (Laragen, Culver City, CA). Cells were maintained in DMEM (MediaTech, Manassas, VA) supplemented with 10% FBS, antibiotic-antimycotic (Life Technologies, Carlsbad, CA) and 2.5 μg/ml plasmocin (Invivogen, San Diego, CA). Cell lines were cultured at 37ºC and 5% CO_2_ in a humidified atmosphere. The following CDK inhibitors were used: PD0332991, SNS032 (Selleck Chemicals, Houston, TX) and dinaciclib (MedKoo Biosciences, Chapel Hill, NC). Cisplatin was purchased from Sigma-Aldrich (St. Louis, MO).

### Plasmids, lentiviral constructs and siRNA

Cyclin E1 cDNA (Addgene, Cambridge, MA, [42]) was cloned into the mammalian expression vector pBABE-puro (Addgene) at the EcoRI site to generate pBABE-CCNE1. Target cells (HEY, and OVCA433) were retrovirally transduced with pBABE-CCNE1 or empty vector. Infected cells were selected with 5 μg/ml puromycin (Gibco, Carlsbad, CA) for 6d prior to use. Lentiviral pLKO constructs for *CCNE1* were a kind gift from Dr. W. C. Hahn (Boston, MA). Inducible pTRIPZ constructs for *ETV5* and *CCNE1* were obtained from ThermoScientific (Waltham, MA; shETV5: catalogue number RHS4741-EG2119, shCCNE1: catalogue number RHS4696-200708509). Transfection of siRNA was performed with Lipofectamine 2000 (Invitrogen, Carlsbad, CA) in OptiMEM (Life Technologies).

### Western blot analysis

Western blot and protein detection was performed as previously described [43]. The following primary antibodies were used: RB, phospho-RB^S807/S811^, CDC25C, phospho-AKT^S473^, phospho-cyclin E1^T62^, phospho-ERK1/2^T202/Y204^, ERBB2, phospho-ERBB2^Y1221/1222^ and MYC (Cell Signaling, Danvers, MA); BRCA1, p53, cyclin E, CDK1, CDK2, ETV4, ETV5 and HRAS (Santa Cruz Biotechnology, Santa Cruz, CA); Actin (Abcam, Cambridge, MA); GAPDH (Fitzgerald, Acton, MA); β-Actin (Sigma-Aldrich); PAX8 (Epitomics, Burlingame, CA);

### Quantitative RT-PCR (qPCR)

Total RNA isolation, cDNA generation and qPCR were performed as previously described [43]. Primer sequences used for qPCR were obtained from SAB Biosciences (Valencia, CA; Supplemental Information).

### Antibody arrays

Parental and CDKi-resistant cells were treated with the indicated CDKi and cell lysates incubated with phospho-kinase or phospho-MAPK profiler antibody arrays (R&D Systems, Minneapolis, MN). After washing, the membranes were incubated with a cocktail of biotinylated antibodies and streptavidin–HRP according to the manufacturer's protocol (R&D Systems). Proteins were detected using chemiluminescence.

### Chromatin immunoprecipitation (ChIP)

ChIP assay was performed on naïve SKOV3, SKOV3-PD/SNS^R^-1 early, and SKOV3-PD/SNS^R^-1 late cells using the SimpleChIP Enzymatic Chromatin IP Kit according to the manufacturer's protocol (Cell Signaling). Briefly, cells were cross-linked with 1% formaldehyde in culture medium at room temperature for 10 minutes. An aliquot to serve as input DNA was set aside. After quenching with glycine and subsequent washes, chromatin was digested by micrococcal nuclease and sonication. Sheared chromatin-protein complexes were incubated with antibodies against IgG and E2F1 (Santa Cruz Biotechnology) overnight at 4°C. Complexes were immunoprecipitated using ChIP grade Protein G Magnetic Beads. After washes, DNA was eluted from the beads by incubation for 30 minutes at 65°C. Cross-links were reversed by incubation with Proteinase K overnight at 65°C. DNA was purified using spin columns. ChIP DNA was then subjected to qPCR using promoter primers for *BRCA1*, *BRCA2*, *CCND1*, and *ETV5* (SA Biosciences; Supplemental Information) and the iQ SYBR-Green Supermix (BioRad, Hercules, CA). The qPCR reaction was performed using the CFX96 Real-Time System (BioRad).

### Proliferation assays

To assay viability, cells (1 × 10^3^ per well) were plated in 96-well plates. After an overnight incubation, cells were harvested at 0 h (T0) using a luminometric assay performed according to the manufacturer's protocol (CellTiter-Glo Luminescent Cell Viability Assay, Promega, Madison, WI) or harvested after treatment with SNS032, PD0332991, dinaciclib or cisplatin for the indicated times and concentrations. Luminescence was measured on a Veritas microplate luminometer (Turner BioSystems, Sunnyvale, CA) after 15 min. Relative viability was calculated as reported before [10]. IC_50_ values were determined using GraphPad Prism version 5 software (San Diego, CA). Proliferation rate was determined by seeding and harvesting cells at T0 exactly as described above with subsequent harvests performed daily for five consecutive days or after 6d (T6). Proliferation rate was also determined by seeding 5 × 10^5^ cells per 100-mm (T0) followed by hemocytometer cell counting after 4-6d. Seeding of cells and cell counting were repeated at regular intervals for up to 14 d. Cell viability was also assayed by crystal violet staining. Briefly, cells (5 × 10^5^ per 100-mm plate or 1 × 10^5^ per 60-mm plate) were seeded and treated as indicated. Cells were fixed in 4% formaldehyde, washed and subsequently stained with 0.1% crystal violet. Quantitation was performed by extracting the crystal violet dye with 10% acetic acid and measuring the absorbance at 550 nm on an Ultramark EX Microplate spectrophotometer (BioRad). Anchorage-independent cell proliferation was determined by soft agar assay as previously described [10]. At least two independent experiments were performed in triplicate for each cell line for all proliferation assays.

### Flow cytometry

Cells (5 × 10^5^) were plated into 100-mm tissue culture plates. After an overnight incubation SNS032, PD0332991 and dinaciclib were added at the indicated concentrations and samples were harvested 48 h post-treatment. Cell cycle analysis was performed by flow cytometry after BrdU incorporation and propidium iodide (PI) staining using standard methods (BD Biosciences, San Jose, CA). Briefly, samples were labeled with BrdU (Amersham, Buckinghamshire, UK) for 2 h, washed with PBS then fixed in 70% ice-cold ethanol. After an overnight incubation at 4°C, the cells were treated with 2 N HCl/0.5%Triton X100 then treated with borate neutralizing solution. BrdU-labeled cells were detected with anti-BrdU (BD Biosciences) and FITC-labeled secondary antibody (Vector Laboratories, Burlingame, CA). Cells were subsequently treated with PI (Sigma-Aldrich) and RNase A (Sigma-Aldrich). Samples were analyzed for BrdU/PI incorporation with a Becton Dickinson FACScan (Franklin Lakes, NJ) using CellQuest version 3.1 software (BD Biosciences) to gate G_1_, S and G_2_-M cells. The results generated were from multiple independent experiments performed in triplicate. A total of 10,000 events were collected for final analysis.

### Senescence β-galactosidase staining

Cells (5 × 10^5^) were plated into 10 cm tissue culture plates and treated with PD0332991 for 4 weeks while being passaged repeatedly. Cellular senescence was assessed using the Senescence *β*-galactosidase staining kit as per manufacturer's instructions (Cell Signaling).

### Array CGH

Genomic DNA was extracted using the DNeasy Tissue Kit as per manufacturer's instructions (Qiagen, Valencia, CA). Pooled female genomic DNA (Promega, Madison, WI) was differentially labeled as a control sample. Hybridization was performed on 135,000 oligonucleotide arrays (Roche-Nimblegen, Madison, WI) with an average probe spacing every 35 kb according to manufacturer's recommendations. Arrays were imaged using a Genepix 4000B Scanner with GenePixPro software (Molecular Devices, Sunnyvale, CA), and data was analyzed with the NimbleScan and Genoglyphix software (Roche-Nimblegen). Variants were defined as segments that have at least 5 probes and a minimum log ratio of 0.3, 200 probes and a log ratio of 0.2 or 500 probes and a log ratio of 0.1. Variants were compared against public databases, including the Database of Genomic Variants (The Hospital for Sick Children, Toronto, Canada) and the Copy Number Variation Project at the Children's Hospital of Philadelphia to benign population variants. Polyclonal populations of naïve and CDKi-resistant cells were analyzed.

### Microarray gene expression profiling

Total RNA samples were assessed for concentration using Thermo Scientific's NanoDrop 8000 Spectrophotometer (Carlsbad, CA) and degradation using the Agilent RNA 6000 Nano Kit with the Agilent 2100 Bioanalyzer (Santa Clara, CA). 250 ng of total RNA was amplified and biotinylated with Ambion's Illumina® TotalPrep™ RNA Amplification Kit (Austin, TX) for hybridization to Illumina HumanHT-12 v4 Expression BeadChip (La Jolla, CA) per manufacturer's instructions. Expression profiling was performed on two individual cell clones per group and profiles were deposited in the GEO repository (accession number GSE63529).

### Microarray data normalization and analysis

47,231 probes were queried on microarray. Raw intensities were quantile-normalized with background subtraction, no imputation of data of missing beads. To minimize the effect of background noise on calculations, a floor or threshold was established so that all raw probe intensities below the median array intensity value of 11 were increased to 11. 10,989 probes that were not significantly expressed over background (p-value > 0.05) for all samples were removed from further analysis. 170 additional probes without gene names were removed (cDNA clones and “hypothetical” mRNAs). Significantly differentially expressed genes were identified using a Wilcoxon *t*-test between groups of samples and a minimum of +/− 1.4 fold change between groups. q-value's were calculated using the method of Benjamini and Hochberg [44].

### Supervised hierarchical clustering

A known E2F target gene list was used to evaluate the samples for gene regulation. In order to look for coordinated E2F gene regulation, the samples and the gene intensities of the (80 unique gene IDs, 117 probes) 80 E2F genes were subjected to two-way hierarchical clustering. All gene expression data were log_2_ transformed. A distance matrix using Pearson correlations was generated with both samples and genes and used to plot gene and sample dendrograms representing the correlation or distance between samples and genes. The heat map was generated using Z-scores to better visualize differential gene expression using gplots package (gplots version 2.12.1) in R version 3.0.2.

### *In Vivo* tumor xenograft studies

Female nude mice obtained from Charles River Laboratories (Wilmington, MA) were injected subcutaneously in both flanks with 1 × 10^6^ HEY cells or 1.5 × 10^6^ OVCAR3 cells supplemented with 50% v/v matrigel (Becton Dickinson) to optimize OVCAR3 tumor uptake and growth. When tumors reached 100 mm^3^, mice were randomly divided into either 3 treatment groups (HEY tumor xenografts) or 4 treatment groups (OVCAR3 tumor xenografts). Mice injected with HEY cells were treated intraperitoneally twice weekly with 50 mg/kg PD0332991, 15 mg/kg SNS032 or vehicle. Mice injected with OVCAR3 cells were treated intraperitoneally once weekly with 40 mg/kg dinaciclib, 2 mg/kg cisplatin, combination of dinaciclib and cisplatin (on separate days) or vehicle. Tumors were measured by caliper at regular intervals and tumor volume was calculated using the formula *(W)^2^ × L/2*. Mice were maintained and euthanized according to IACUC guidelines.

### Statistical analysis

Data were analyzed using a Student's *t*-test.

## SUPPLEMENTARY MATERIALS, TABLES AND FIGURES




