# Supplementary material for: Cyclin E1 and RTK/RAS signaling drive CDK inhibitor resistance via activation of E2F and ETS
Source: Oncotarget. 2014 Dec 22;6(2):696–714. doi: 10.18632/oncotarget.2673 (PMC4359249; doi:10.18632/oncotarget.2673)
Supplement: Supplementary file 1 [file oncotarget-06-696-s001.pdf]

SUPPLEMENTARY MATERIALS, TABLES AND FIGURES

Primers used for qPCR

All primer sets used for qPCR were purchased from SA Biosciences. Primers targeting the following genes were used for gene expression analysis: *BRCA1*, *BRCA2*, *CCND2*, *CCNE1*, *CDK1*, *ERBB2*, *ETV4*, *ETV*5 and *PLK2*. Genomic primers targeting the following promoters were used for ChIP assays (locations of primers relative to the promoter start site are given in parentheses): *BRCA1* (-1K and +1K), *BRCA2* (-2K), *CCND2* (+1K) and *ETV5* (-2K and -1K). Genomic primers targeting the following genes were used for copy number validation: *CCNE1*, *RB1*, *ERBB2* and *MYC*.

Drug combination studies

Synergy between dinaciclib and cisplatin was determined as previously described [43]. The drug combination effect of dinaciclib and cisplatin was calculated using the non-constant ratio method as measured by the combination index (CI) using Calcusyn software (Biosoft, Cambridge, UK) [45]. A CI < 0.9, CI = 0.9-1.1 and CI > 1.1 indicate antagonism, additive effect and synergism, respectively.

Supplementary Table S1. CDK selectivity of PD0332991, SNS032 and dinaciclib according to published data.

(nM)	CDK1	CDK2	CDK4	CDK6	CDK5	CDK7	CDK9	Reference
PD0332991			11	16				Fry et al., 2004 [7]
SNS032		38				62	4	Misra et al., 2004 [21]
Dinaciclib	3	1			1		4	Parry et al., 2010 [12]

Supplementary Table S2. Copy number and mutation status of HGSOC signature genes and PD0332991 sensitivity in 10 ovarian cancer cell lines. AMP, amplification; MUT, mutation; DEL, deletion; ND, not determined. IC_{50} values of PD0332991 were previously determined by Konecny *et al*. [9]. Copy number and mutation status were verified using the following sources: [1]Konecny *et al*. 2011 [9]; [2]canSAR database; [3]Domcke *et al*. 2013 [46]; [4]our own array CGH data: copy number gain (log_2 ratio > 0.3), amplification (log_2 ratio > 1).

	RB1	CCNE1	CDKN2A/B	p53	MYC	PD0332991 IC_{50} [1] (μM)
CAOV3	LOSS [1,2]		LOSS [1]	MUT [1,3]		> 10
OVCAR3	LOSS [1,2]	AMP [1,2]		MUT [1,2]		> 10
OVCAR4	LOSS [2,4]	GAIN [2]		MUT [2,3]	GAIN [2]	ND
OV90		GAIN [1]	LOSS, MUT [2]	MUT [1,2,3]		> 10
OAW28	LOSS [1,2]			MUT [1,2,3]		> 10
SKOV3			DEL [1,2]	MUT [1,2]		< 0.25
OVCA420				MUT [2]		< 0.25
OVCA433						ND
HEY			DEL [1]		GAIN [4]	< 0.25
DOV13			DEL [1]			< 0.25

Supplementary Table S3: Genome-wide analysis of DNA copy number changes in naïve and CDKi-resistant OAW28, OV90 and SKOV3 as determined by aCGH. Segmented log_2 values are shown.

Supplementary Table S4. Expression profiling of naïve and CDKi-resistant OV90 cells. Differentially expressed E2F target genes (fold change ≥ 1.4 or ≤ -1.4, $p \leq 0.05$) are shown (CDKi-resistant cells versus naïve).

OV90–SNS^{R}

Feature ID	Symbol	Fold Change	*P*-value
**E2F targets**			
ILMN_2374778	DUT	−1.86	0.04
ILMN_2386100	BUB3	−1.71	0.05
ILMN_1706958	PCNA	−1.71	0.05
ILMN_1658143	RFC3	−1.57	0.02
ILMN_1653761	MYCN	1.58	0.05

OV90–PD/SNS^{R}

Feature ID	Symbol	Fold Change	*P*-value
**E2F targets**			
ILMN_2374778	DUT	−2.25	0.03
ILMN_1798210	E2F7	1.42	0.05
Other genes			
ILMN_2294431	CDC25C	−2.01	0.03

Supplementary Table S5. Expression profiling of naïve and CDKi-resistant SKOV3 cells. Differentially expressed E2F target genes (fold change ≥ 1.4 or ≤ -1.4, $p \leq 0.05$) are shown, individually comparing SKOV3-SNS^R, SKOV3-PD/SNS^R early and SKOV3-PD/SNS^R late cells with naïve SKOV3. The last sheet (SKOV3-PD/SNS^R release) compares SKOV3-PD/SNS^R release cells with SKOV3-PD/SNS^R late cells.

SKOV3–SNS^R

Feature ID	Symbol	Fold Change	*P*-value
**E2F targets**			
ILMN_2157099	CCNA1	−6.94	0.01
ILMN_1798654	MCM6	−1.93	0.02
ILMN_1651237	CDT1	−1.85	0.01
ILMN_1736176	PLK1	−1.75	0.02
ILMN_3234884	KIF22	−1.5	0.02
ILMN_1734827	MKI67	−1.42	0.05
ILMN_1795852	CCNE1	1.44	4.31E-03
ILMN_1716895	RPA3	1.48	0.04
ILMN_2379560	CDC14B	1.52	0.03
ILMN_2411190	SMC2	1.58	0.03
**Other genes**			
ILMN_1677429	TWIST2	−4.36	0.05
ILMN_1669523	FOS	−2.02	0.03
ILMN_1664010	ELF1	−1.82	0.05
ILMN_2352131	ERBB2	1.36	0.05
ILMN_2364529	EZH2	1.61	0.04
ILMN_1672908	TWIST1	1.75	6.59E-03
ILMN_1798975	EGFR	2.22	8.58E-03
ILMN_2325610	AKT3	4.43	0.02

SKOV3–PD/SNS^R early

Feature ID	Symbol	Fold Change	*P*-value
**E2F targets**			
ILMN_2157099	CCNA1	−6.64	5.70E-03
ILMN_1696591	RB1	−2.55	8.64E-03
ILMN_1651237	CDT1	−1.89	0.02
ILMN_1661717	TFDP1	−1.57	0.05
ILMN_2384785	CCNE1	1.25	0.02
ILMN_1756326	CKS2	1.48	6.50E-04
**Other targets**			
ILMN_1677429	TWIST2	−5.46	0.05
ILMN_1664010	ELF1	−2.64	8.27E-03
ILMN_1772486	ELF2	−1.61	0.05
ILMN_2352131	ERBB2	1.47	0.04

(*Continued*)

SKOV3–PD/SNS^R early (*Continued*)			
**Feature ID**	**Symbol**	**Fold Change**	***P*-value**
ILMN_2338323	CDC25B	1.52	0.03
ILMN_2364529	EZH2	1.57	0.03
ILMN_1672908	TWIST1	2.28	0.02
ILMN_1739222	ETV5	2.75	0.05
ILMN_2341661	ETV4	3.31	0.05
ILMN_2325610	AKT3	5.6	0.02

SKOV3–PD/SNS^R late			
**Feature ID**	**Symbol**	**Fold Change**	***P*-value**
**E2F targets**			
ILMN_2157099	CCNA1	−8.02	4.90E-03
ILMN_1696591	RB1	−2.55	2.34E-03
ILMN_1696713	POLA2	1.4	0.03
ILMN_1686097	TOP2A	1.4	0.05
ILMN_2120965	NPAT	1.47	0.02
ILMN_2191436	POLA1	1.47	0.01
ILMN_1670238	CDC45L	1.48	4.59E-03
ILMN_1785914	CDC6	1.48	0.03
ILMN_1665559	CDK2	1.48	7.88E-03
ILMN_1753582	RPA2	1.5	0.02
ILMN_1651237	CDT1	1.51	0.01
ILMN_1687074	CDC14B	1.54	0.03
ILMN_1724489	RFC4	1.54	1.81E-03
ILMN_2374778	DUT	1.62	3.41E-03
ILMN_1681503	MCM2	1.64	0.03
ILMN_1688480	CCND1	1.71	0.03
ILMN_1716895	RPA3	1.73	1.39E-03
ILMN_1756326	CKS2	1.75	3.27E-03
ILMN_1795852	CCNE1	1.84	1.19E-03
ILMN_1799667	KIF4A	1.88	0.02
ILMN_1663195	MCM7	1.88	4.23E-03
ILMN_1668721	CCND3	2.06	3.02E-03
ILMN_1777233	E2F2	2.07	1.13E-03
ILMN_2412384	CCNE2	2.09	0.03
ILMN_1678669	RRM2	2.2	7.71E-03
ILMN_1652580	POLD1	2.22	0.02
ILMN_2202948	BUB1	2.26	5.10E-05
ILMN_1655733	RFC2	2.54	1.70E-03
ILMN_1684217	AURKB	3.1	0.03

(*Continued*)

SKOV3–PD/SNS^R late (*Continued*)			
**Feature ID**	**Symbol**	**Fold Change**	***P*-value**
ILMN_1711005	CDC25A	4.91	3.95E-03
**Other genes**			
ILMN_1664010	ELF1	−3.31	7.01E-03
ILMN_1655740	SNAI2	−2.57	1.02E-03
ILMN_2086077	JUNB	−2.26	0.05
ILMN_1844692	FOXO3	−2.04	1.89E-03
ILMN_1691559	ELF2	−1.61	0.03
ILMN_2338323	CDC25B	1.46	0.02
ILMN_2352131	ERBB2	1.5	0.02
ILMN_2257432	RAD51	1.65	7.38E-03
ILMN_2344971	FOXM1	1.77	0.02
ILMN_2357438	AURKA	1.87	2.69E-03
ILMN_1739222	ETV5	1.87	2.70E-03
ILMN_1672908	TWIST1	1.97	1.70E-03
ILMN_2364529	EZH2	2.18	2.08E-03
ILMN_2341661	ETV4	3.49	8.82E-03
ILMN_2325610	AKT3	10.47	8.30E-03

SKOV3–PD/SNS^R release			
**Feature ID**	**Symbol**	**Fold Change**	***P*-value**
**E2F targets**			
ILMN_2110908	MYC	−1.51	0.02
ILMN_1669502	E2F3	−1.45	0.03
ILMN_1736176	PLK1	1.4	0.05
ILMN_1663195	MCM7	1.47	6.31E-04
ILMN_1658143	RFC3	1.52	0.01
ILMN_1785914	CDC6	1.54	0.05
ILMN_3234884	KIF22	1.74	0.03
ILMN_1777233	E2F2	1.94	0.03
ILMN_1684217	AURKB	2.11	0.02
ILMN_1651237	CDT1	2.21	0.01
ILMN_1711005	CDC25A	2.57	0.01
ILMN_2296422	SMC2	3.43	0.02
**Other genes**			
ILMN_1739222	ETV5	−3.42	0.04
ILMN_2341661	ETV4	−2.81	0.05
ILMN_2395236	CHEK2	−2.02	0.04
ILMN_2352131	ERBB2	−1.41	0.03
ILMN_1720158	ETS2	1.6	0.05
ILMN_2364529	EZH2	1.65	9.74E-03

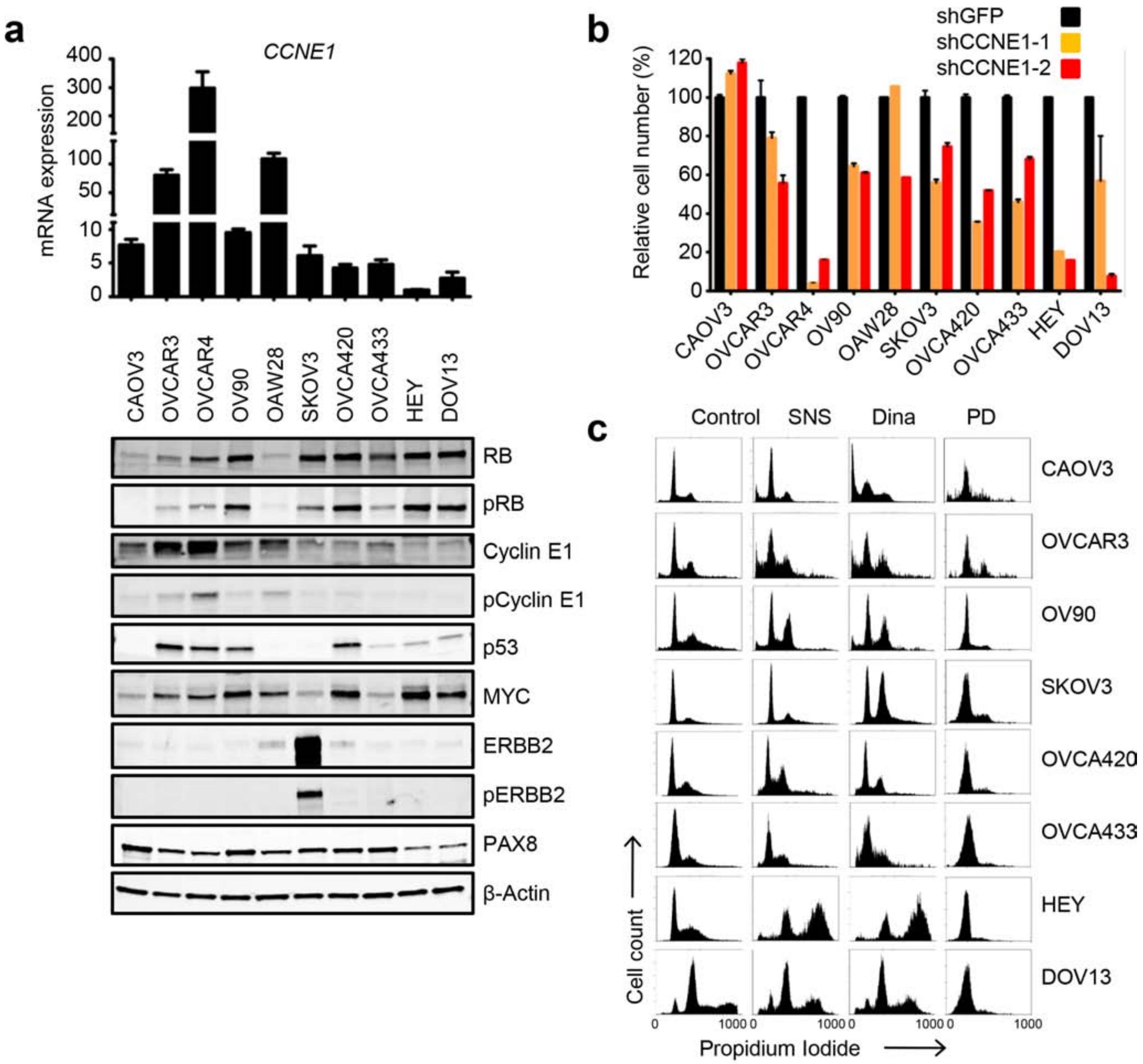

Supplementary Figure S1: CDKi-induced proliferation arrest and downregulation of E2F target genes in different ovarian cancer cell lines. (a) Western blot analysis across a panel of 10 ovarian cancer cell lines with corresponding *CCNE1* RNA expression. Total RNA and whole-cell extracts were isolated from subconfluent cell cultures. Lysates were immunoblotted with the indicated antibodies and *CCNE1* gene expression was determined by qPCR (upper). **(b)** Cyclin E1 is essential for proliferation in most ovarian cancer cell lines. Cell lines were infected with lentiviral shRNAs targeting GFP (control) or *CCNE1* (two individual hairpins were used) and puromycin-selected. Relative cell number was assessed by crystal violet stain. **(c)** CDK2 and CDK4/6 inhibition induce cell cycle arrest. Cells were treated with 0.2 µM SNS032, 0.02 µM dinaciclib or 0.5 µM PD0332991 for 48 h, fixed, stained with PI with subsequent flow cytometric analysis. A representative DNA histogram is shown for each condition.

(*Continued*)

d

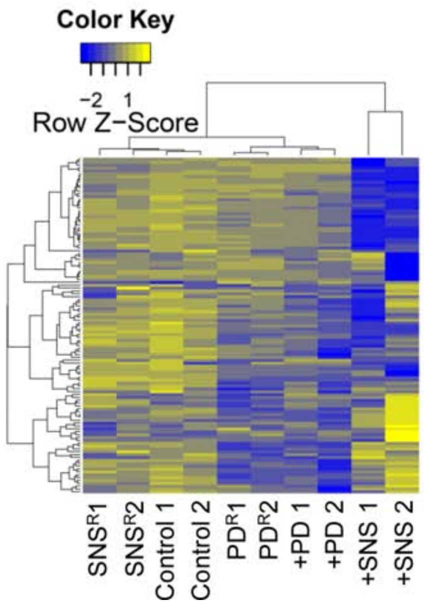

Experiment	Molecular and cellular function	Top regulators	P-value
SNS-treated versus untreated	Cell cycle<br>p-value 4.42E-11 to 4.46E-02<br>350 molecules	1. E2F4<br>2. ERBB2<br>3. TP53	5.53E-10<br>3.69E-06<br>1.02E-05
PD-treated versus untreated	Cell cycle<br>p-value 2.52E-11 to 4.83E-02<br>257 molecules	1. E2F4<br>2. TP53<br>3. E2F1	6.40E-18<br>3.88E-09<br>4.89E-08

Supplementary Figure S1 (*Continued*): (d) Differential regulation of 80 E2F target genes (from Bracken *et al*., 2004 [40]; 117 unique probes) among all samples. Two-way hierarchical clustering using Pearson correlations between samples and genes are represented in dendrograms above and to the right of the heat map. The heat map is colored by Z-score representing higher expression (yellow) and lower expression (blue) than the mean gene expression among all samples. Gene expression profiling followed by Ingenuity Pathway Analysis indicates that cell cycle regulation by E2F and ERBB2 signaling is among the most significantly affected molecular networks in CDKi-treated HEY cells.

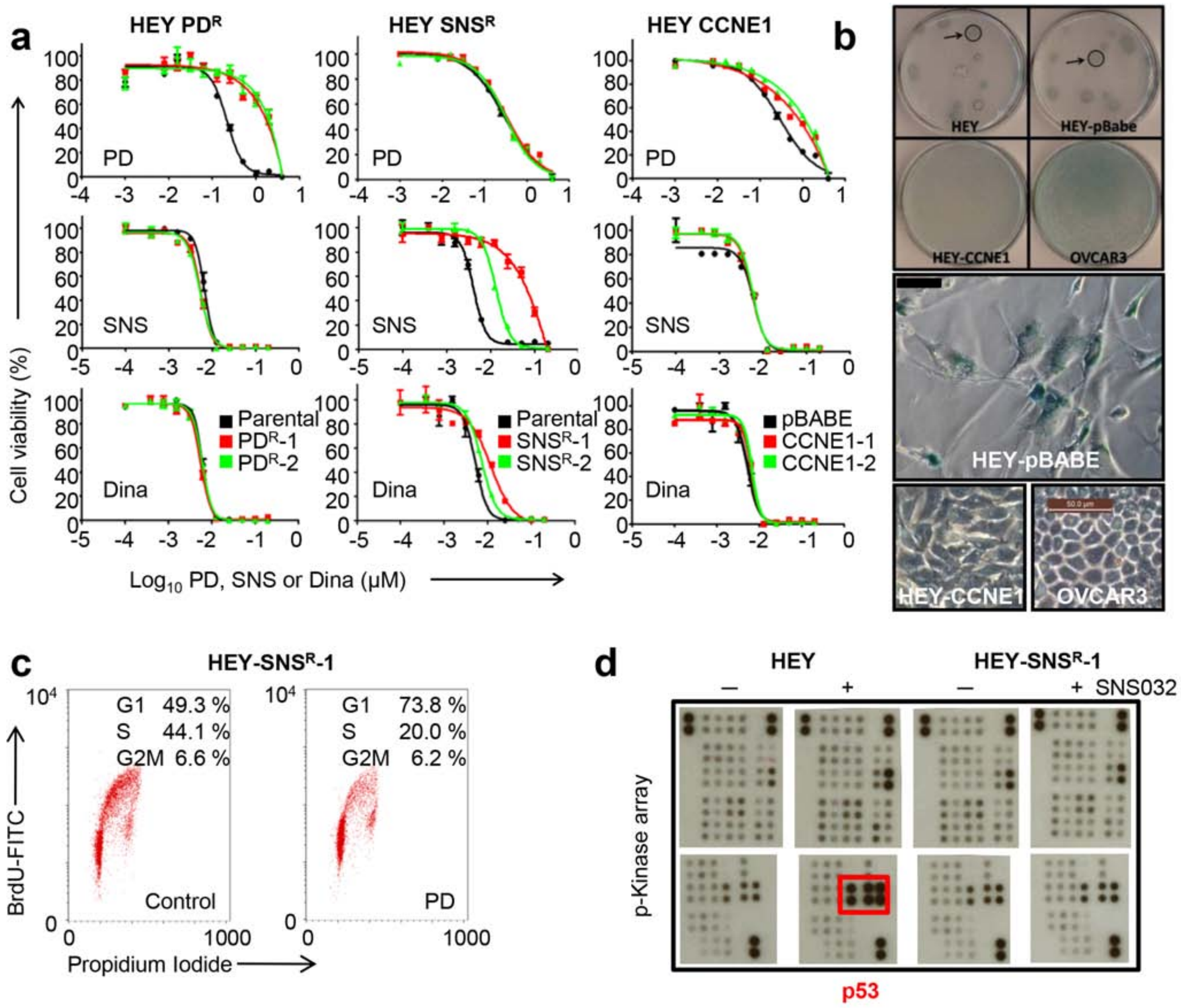

Supplementary Figure S2: CDKi-resistant ovarian cancer cells escape from CDKi-induced proliferation arrest. **(a)** Specific CDKi resistance in HEY-PD^R, HEY-SNS^R and HEY-CCNE1 cells. Cells were grown in the absence or presence of increasing concentrations of PD0332991, SNS032 or dinaciclib for 7d (PD0332991) or 72 h (SNS032, dinaciclib). Cell viability was measured by a luminometric assay. HEY-PD^R cells are resistant to PD0332991 but not to SNS032 or dinaciclib. HEY-CCNE1 cells show partial resistance to PD0332991 but not to SNS032 or dinaciclib. HEY-SNS^R cells are resistant to SNS032 and dinaciclib but not to PD0332991. **(b)** Naïve HEY cells and HEY-pBABE cells undergo senescence upon long term exposure to PD0332991 in low density seeding conditions. HEY-CCNE1 and *CCNE1*-amplified OVCAR3 cells are refractory to PD0332991-induced senescence. Induction of senescence was determined by senescence-associated β-galactosidase staining. Scale bar, 50.0 µm. **(c)** HEY-SNS^R cells remain sensitive to PD0332991. HEY-SNS^R cells were treated with 0.5 µM PD0332991 for 48 h, with subsequent BrdU and PI analysis as described in Fig. 1b. A representative FACS image is shown for each condition. **(d)** HEY-SNS^R cells avoid a p53 response. Parental and HEY-SNS^R cells were treated with or without 0.2 µM SNS032 for 48 h. Cell lysates were analyzed using a human phospho-kinase array. Three spots (in duplicate, boxed) corresponding to three different p53 phosphorylation sites are detected in the SNS032-treated HEY cells but not in HEY-SNS^R cells.

(*Continued*)

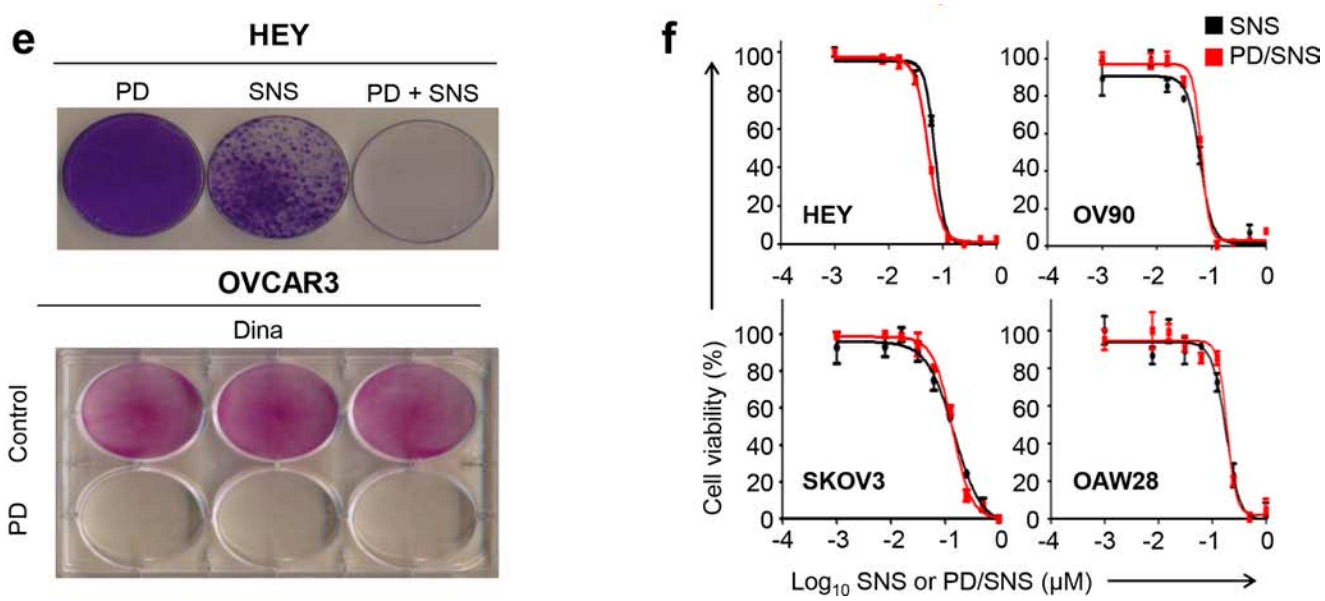

Supplemental Figure S2 (*Continued*): (e) CDK2 inhibition sensitizes ovarian cancer cells to PD0332991. HEY cells were treated with 0.2 µM SNS032, 0.5 µM PD0332991 or combination treatment with 0.2 µM SNS032 and 0.5 µM PD0332991. OVCAR3 cells were treated with 0.02 µM dinaciclib alone or in combination with 0.5 µM PD0332991. Cell viability was determined by crystal violet staining after 3 weeks. **(f)** Acute exposure to combined treatment with PD0332991 and SNS032 exhibits no difference in proliferation compared to treatment with SNS032 alone. Cells were grown in the absence or presence of increasing concentrations of SNS032 alone or equimolar concentrations of both PD0332991 and SNS032 for 72 h. Cell viability was measured by a luminometric assay.

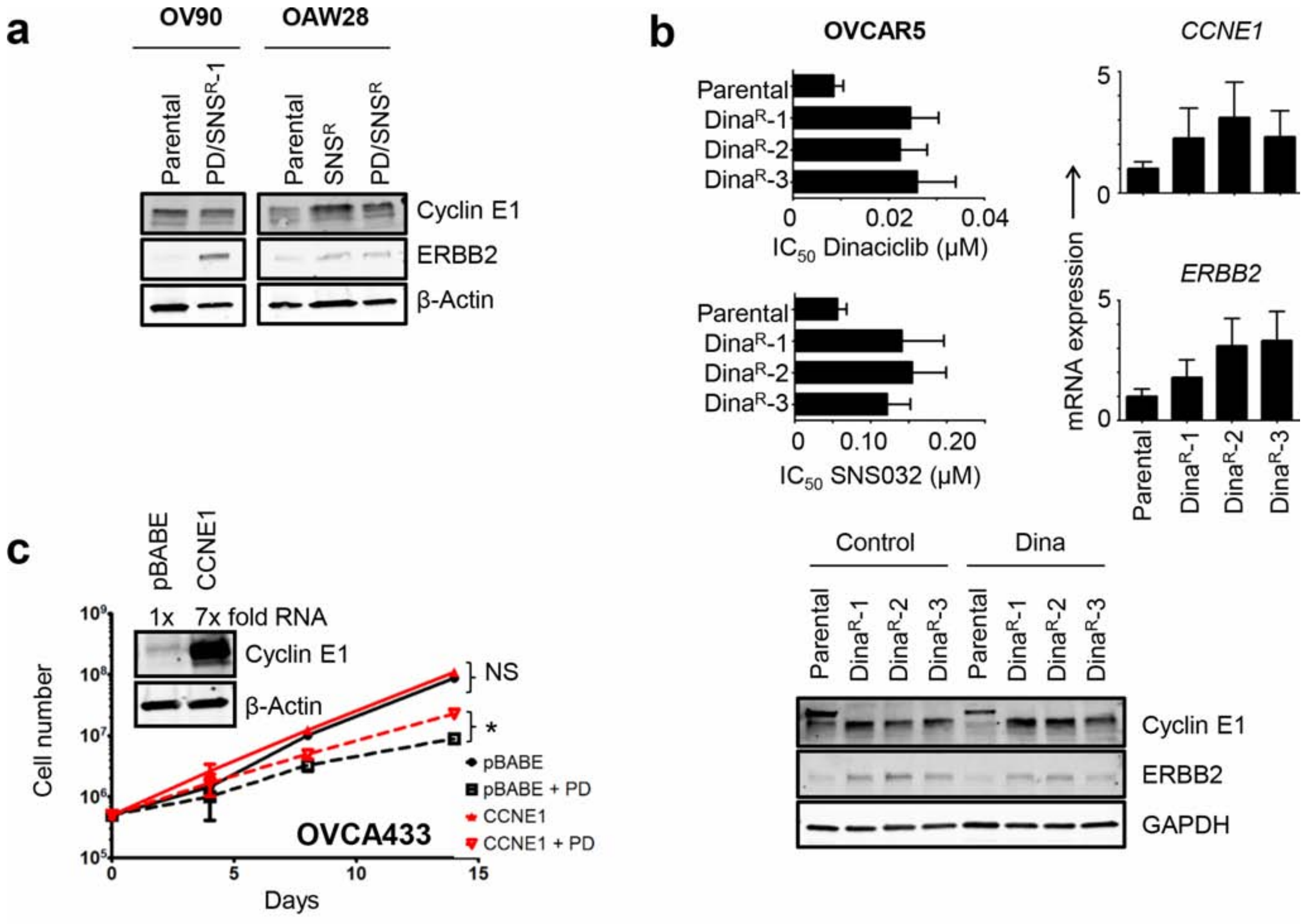

Supplementary Figure S3: Cyclin E1 and ERBB2 confer resistance to CDKi. **(a)** CDKi-resistant OV90 and OAW28 cells induce ERBB2. Protein lysates were probed with antibodies for cyclin E1 and ERBB2. **(b)** OVCAR5 dinaciclib-resistant (Dina^R) cells activate RTK signaling. OVCAR5-Dina^R cells were generated by chronic exposure of OVCAR5 cells to 0.02 µM dinaciclib. Parental and Dina^R cells were treated with increasing concentrations of dinaciclib and IC_{50} values determined using a luminometric viability assay. All Dina^R cell lines conferred resistance to dinaciclib (left), coinciding with increased *CCNE1* and *ERBB2* expression as determined by qPCR (right) and Western blotting (lower). **(c)** OVCA433 cells were stably transduced with retroviral pBABE-CCNE1 or empty vector. Cells were puromycin-selected followed by treatment with 0.5 µM PD0332991 or vehicle for 48 h. Ectopic cyclin E expression was confirmed by Western blot analysis and had no effect on proliferation as measured by cell counting, but conferred resistance to PD0332991. *$p = 0.05$

(*Continued*)

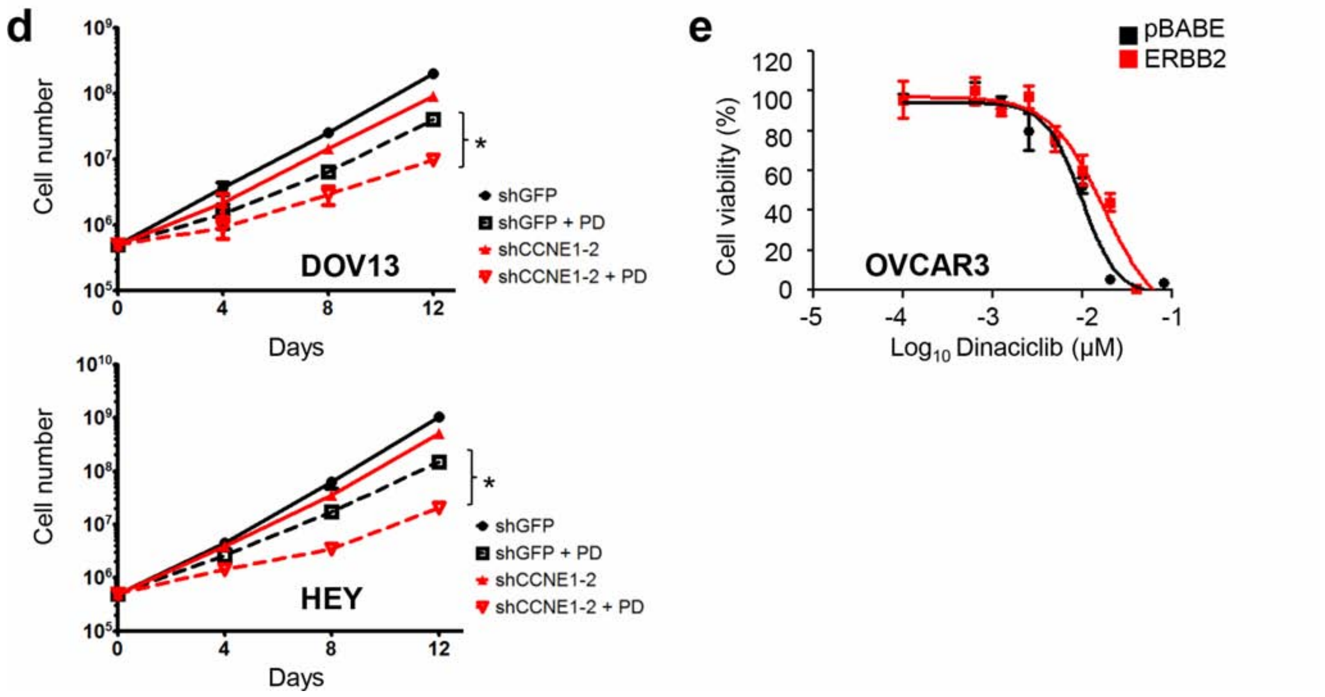

Supplementary Figure S3 (*Continued*): (d) Two individual lentiviral shRNAs targeting CCNE1 were used to genetically deplete cyclin E1 in DOV13 and HEY cells. A shRNA targeting GFP was used as a control. After puromycin selection, cells were seeded for cell proliferation in the absence or presence of 0.5 µM PD0332991. Depletion of cyclin E sensitized cells to PD0332991 as determined by cell counting. Both shCCNE1 clones gave similar results, though only one shCCNE1 clone is shown. *$p = 0.005$ (DOV13), $p = 0.0001$ (HEY) **(e)** Ectopic ERBB2 expression confers resistance to dinaciclib and SNS032. OVCAR3 cells expressing ectopic ERBB2 or empty vector as described in Fig. 3e, were treated with increasing concentrations of dinaciclib and drug sensitivity determined using a luminometric viability assay.

a

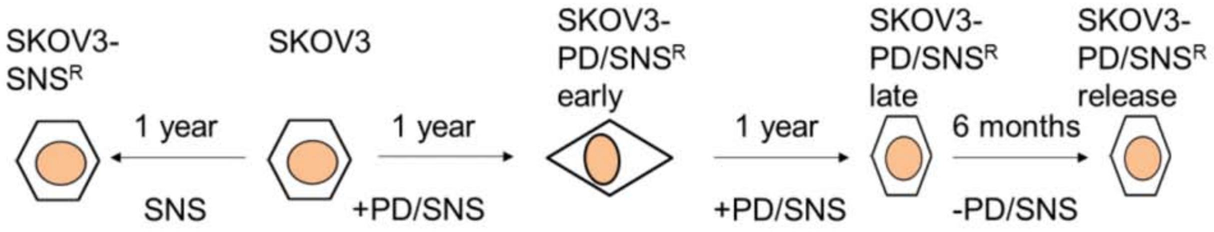

b

	SNS^R	Parental	PD/SNS^R early	PD/SNS^R late	PD/SNS^R release[a]
CCNE1	1.44	1	1.25	1.84	NS
RB1	NS	1	-2.55	-2.55	NS
ETV4	NS	1	3.31	3.49	-2.81
ETV5	NS	1	2.75	1.87	-3.42
AKT3	4.43	1	5.60	10.47	NS
ERBB2	1.36	1	1.47	1.50	-1.41

c

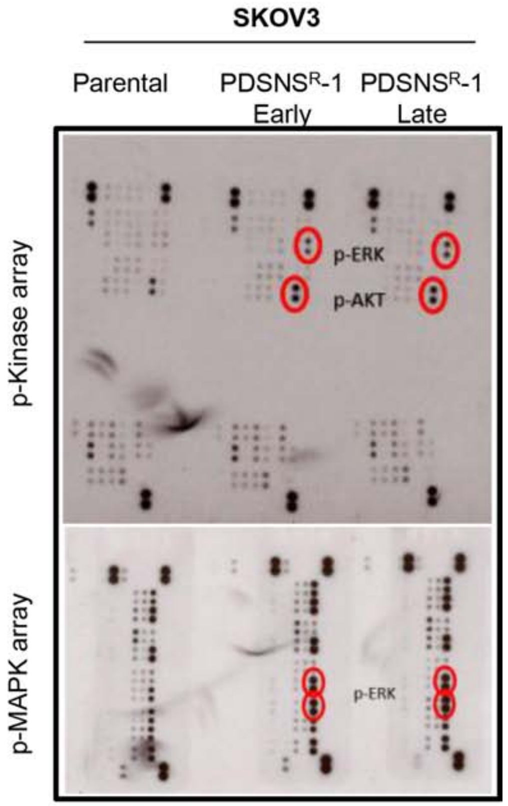

Supplementary Figure S4: Dynamic expression changes and associated phenotypes in CDKi-resistant SKOV3 cells. **(a)** Real time monitoring of CDKi resistance. CDKi-resistant SKOV3 cells were generated by chronic exposure to either 0.2 µM SNS032 alone or a combination of 0.2 µM PD0332991 and 0.2 µM SNS032 for up to two years. SKOV3-PD/SNS^R release cells were analyzed after 6 months in the absence of CDKi following two years of exposure. **(b)** CDKi-resistant SKOV3 cells exhibit RNA expression changes, some of which correlate with *de novo* DNA copy number alterations (*CCNE1*, *RB1*, *AKT3*). In contrast, expression levels of *ERBB2*, *ETV4* and *ETV5* revert back to baseline upon CDKi withdrawal. Expression analysis by microarray was performed in SKOV3-PD/SNS^R (early and late), SNS^R and parental cells. Gene expression changes in the subclones are displayed as fold change compared to the parental control set to 1. [a]Changes in SKOV3-PD/SNS^R release cells were compared to SKOV3-PD/SNS^R late cells as a control set to 1. NS, not significant. **(c)** SKOV3-PD/SNS^R (early and late) and parental cells were analyzed using a human phospho-kinase (upper) and phospho-MAPK (lower) array. Encircled spots (in duplicate) corresponding to phospho-AKT (upper) and phospho-ERK (upper and lower) were detected in SKOV3-PD/SNS^R cells but not in naïve cells.

(*Continued*)

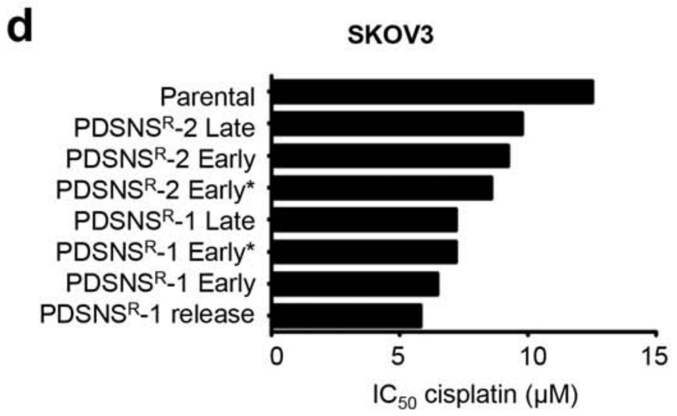

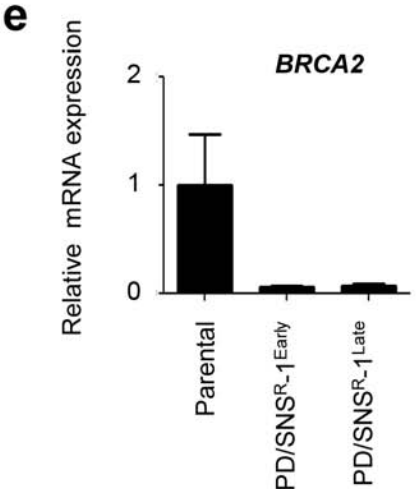

Supplementary Figure S4 (*Continued*): (d) SKOV3-PD/SNS^R and OVCAR5-Dina^R subclones exhibit cisplatin sensitivity. Cells were grown in the absence or presence of increasing concentrations of cisplatin for 48 h and IC_{50} values determined using a luminometric viability assay. *Denotes SKOV3 cells that were exposed to PD0332991/SNS032 for only 3 months compared to 1 yr (early) and 2 yr (late). **(e)** *BRCA2* is downregulated in CDKi-resistant SKOV3 cells. Gene expression in SKOV3-PD/SNS^R and parental SKOV3 cells was determined by qPCR. Downregulation of *BRCA2* correlates with genomic loss of *BRCA2* as part of the chromosome 13 region that harbors *RB1*.

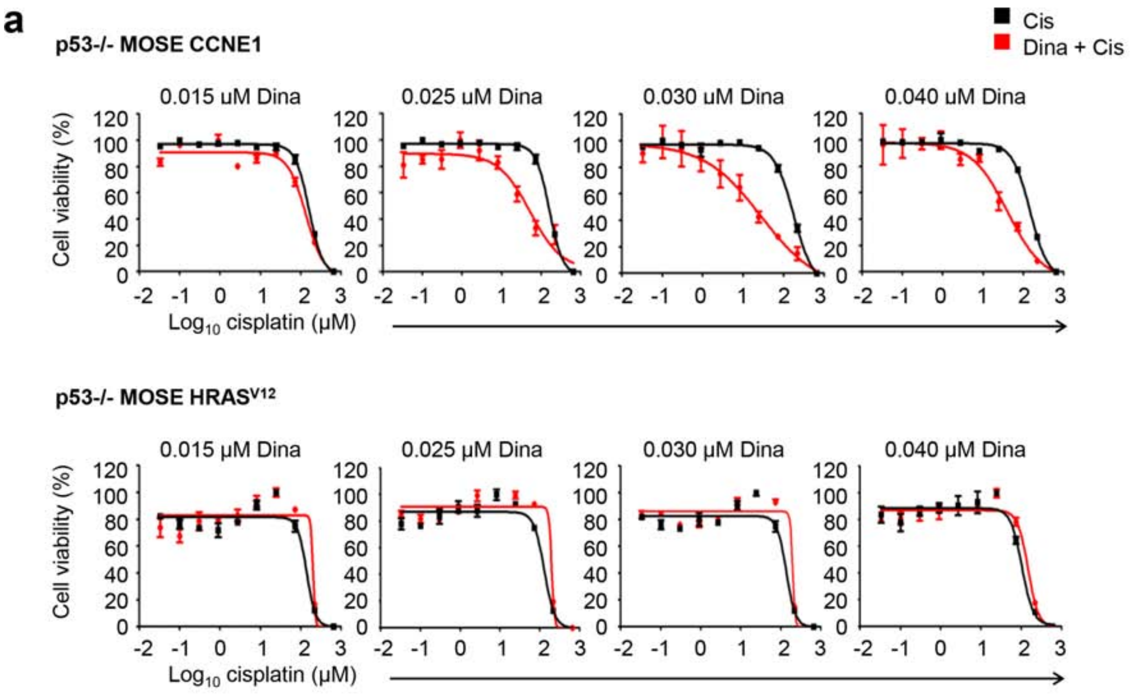

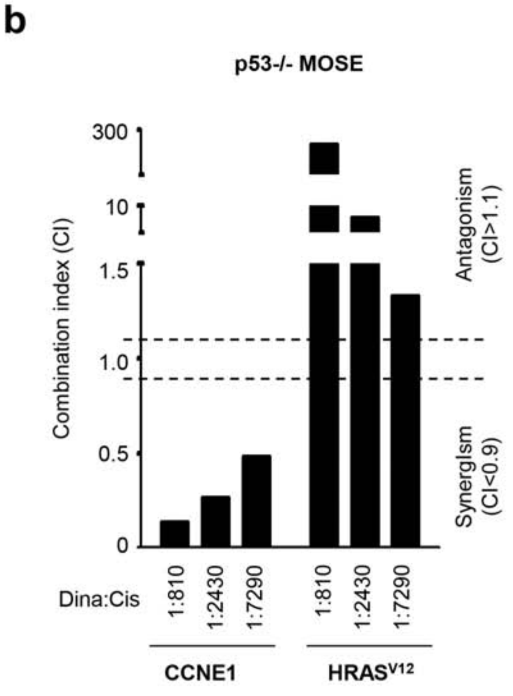

c

	Colony number ±SD Cis	Cis/Dina
**SKOV3**		
1 µM Cis	>100	>100
2 µM Cis	>100	>100
5 µM Cis	>100	>100
10 µM Cis	>100	>100
**OVCAR4**		
1 µM Cis	>100	>100
2 µM Cis	>100	>100
5 µM Cis	1.0 ±1.7	0.3 ±0.6
10 µM Cis	0	0
**OAW28**		
1 µM Cis	>100	>100
2 µM Cis	7.0 ±2.0	0.7 ±1.2
5 µM Cis	4.0 ±3.0	0.7 ±1.2
10 µM Cis	0	0
**OVCAR3**		
1 µM Cis	17.7 ±3.8	5.3 ±2.3
2 µM Cis	6.0 ±1.0	0.3 ±0.6
5 µM Cis	0	0.3 ±0.6
10 µM Cis	0	0

Supplementary Figure S5: Dinaciclib sensitizes cyclin E1-driven cancers to cisplatin. (a) Similar to Fig. 6c, p53-/- MOSE-CCNE1 and p53-/- MOSE-HRAS cells were grown in the absence or presence of fixed concentrations of dinaciclib (0.015 µM-0.04 µM) for 24 h with increasing concentrations of cisplatin added for an additional 48 h. Cell viability was measured by a luminometric assay. **(b)** Drug combination effect of dinaciclib and cisplatin was determined using Calcusyn software. Applying the non-constant ratio method, a fixed concentration of dinaciclib (0.03 µM) and three cisplatin concentrations, 24.3 µM, 72.9 µM and 218.7 µM corresponding to dinaciclib:cisplatin ratios of 1:810, 1:2430 and 1:7290, respectively, were used to determine combination index (CI) values. The cisplatin concentrations selected represent a range of doses that showed increased sensitivity to dinaciclib in p53-/- MOSE CCNE1 cells but not in MOSE HRAS^{v12} (Fig. 6c). CI > 1.1, CI = 0.9-1.1 and CI > 1.1 indicate antagonism, additive effect and synergism, respectively. Similar results were observed using different fixed concentrations of dinaciclib, though not shown. **(c)** Cells were grown in 10nM dinaciclib, cisplatin (1, 2, 5 or 10 µM) or a combination of dinaciclib and cisplatin. After two weeks cells were stained for crystal violet and quantitated.
